# A genome-wide association study in a large community-based cohort identifies multiple loci associated with susceptibility to bacterial and viral infections

**DOI:** 10.1038/s41598-022-05838-z

**Published:** 2022-02-16

**Authors:** Thomas Tängdén, Stefan Gustafsson, Abhiram S. Rao, Erik Ingelsson

**Affiliations:** 1 Infection Medicine, Department of Medical Sciences, Uppsala University, Uppsala, Sweden; 2 Molecular Epidemiology and Science for Life Laboratory, Department of Medical Sciences, Uppsala University, Uppsala, Sweden; 3 Division of Cardiovascular Medicine, Department of Medicine, Stanford University School of Medicine, Stanford, CA 94305 USA; 4 Stanford Cardiovascular Institute, Stanford University, Stanford, CA 94305 USA; 5 Stanford Diabetes Research Center, Stanford University, Stanford, CA 94305 USA

**Keywords:** Genome-wide association studies, Infectious diseases

## Abstract

There is limited data on host-specific genetic determinants of susceptibility to bacterial and viral infections. Genome-wide association studies using large population cohorts can be a first step towards identifying patients prone to infectious diseases and targets for new therapies. Genetic variants associated with clinically relevant entities of bacterial and viral infections (e.g., abdominal infections, respiratory infections, and sepsis) in 337,484 participants of the UK Biobank cohort were explored by genome-wide association analyses. Cases (n = 81,179) were identified based on ICD-10 diagnosis codes of hospital inpatient and death registries. Functional annotation was performed using gene expression (eQTL) data. Fifty-seven unique genome-wide significant loci were found, many of which are novel in the context of infectious diseases. Some of the detected genetic variants were previously reported associated with infectious, inflammatory, autoimmune, and malignant diseases or key components of the immune system (e.g., white blood cells, cytokines). Fine mapping of the HLA region revealed significant associations with HLA-DQA1, HLA-DRB1, and HLA-DRB4 locus alleles. *PPP1R14A* showed strong colocalization with abdominal infections and gene expression in sigmoid and transverse colon, suggesting causality. Shared significant loci across infections and non-infectious phenotypes in the UK Biobank cohort were found, suggesting associations for example between SNPs identified for abdominal infections and CRP, rheumatoid arthritis, and diabetes mellitus. We report multiple loci associated with bacterial and viral infections. A better understanding of the genetic determinants of bacterial and viral infections can be useful to identify patients at risk and in the development of new drugs.

## Introduction

Bacterial and viral infections are common causes of mortality worldwide^1,2^. As effective antimicrobial treatment is increasingly threatened by the spread of resistant pathogens, new strategies and alternative therapies must be explored to reduce the incidence and burden of these infections^3^. The epidemiological situation, exposure and virulence of the invading pathogen are important to determine the risk of acquiring transmittable diseases, such as respiratory tract infections (RTIs)^4^. Many patient-specific factors, such as older age, malignancies, chronic diseases, and immunosuppression, are also known to increase the incidence and severity of viral and bacterial infections^5,6^. Understanding the risk factors for infections is important in clinical practice to guide strategies for prevention and treatment.

Genome-wide association studies (GWAS) in large population cohorts whereby associations between phenotypes and genetic variants across the whole genome are examined is a powerful tool to discover genetic determinants of disease and to uncover novel biology^7^. To date, such data is scarce for infections, and consequently, the genetic variants predisposing for these diseases are largely unknown. A previous GWAS based on self-reported history of common infections in > 200,000 individuals identified multiple loci associated with disease, most notably in genes related to the immune response^8^. These results, along with studies of heritability of infectious diseases^9^, suggest that genetic determinants play an important role for patient susceptibility to bacterial and viral infections. Exploring such undiscovered host-specific factors could be important for detection of patients at increased risk of infection and provide an avenue to identify targets for new drugs.

In the present study, we assessed the impact of genetic variation on the incidence of bacterial and viral infectious diseases in a cohort of ~ 350,000 individuals using results of genome-wide genotype data and diagnose codes from hospital inpatient and death registries.

## Methods

### Participants and phenotypes

Data on genetic variation and incidence of infections were obtained for participants of white British ancestry from the UK Biobank longitudinal community-based cohort study. The UK Biobank protocol has been described previously^10^ and is available online at https://www.ukbiobank.ac.uk. In short, approximately 500,000 individuals aged 40–69 years were included in the study at multiple sites in the United Kingdom during 2006–2010. The participants are monitored with regard to lifestyle, health conditions and biomarkers.

This study included 337,484 UK Biobank participants (mean age of 57 years), of which 181,236 (53.7%) were female and 156,248 (46.3%) were male. Infections were defined using hospital inpatient data and causes of death based on the International Classification of Disease (ICD)-10 codes (Version: 2015, available at https://www.who.int). Clinically relevant entities, such as abdominal infections, RTIs and urinary tract infections (UTIs), were defined by review of all available ICD-10 codes. Since most acute infection episodes do not require more than one health-care visit, a single ICD-10 diagnose code on one occasion was sufficient for inclusion. Diagnoses indicating suspected or proven bacterial or viral infections were categorized to the different phenotype groups based on anatomical sites and pathogens (Table S1). In some cases, the same ICD-10 diagnose code was included in more than one phenotype. For example, B26 mumps was included in viral infections and B26.2 mumps encephalitis was included in central nervous infections. Both primary and secondary diagnosis codes were considered in the analysis of phenotype-genotype associations, and all individuals with an infectious disease event (before or after baseline) were considered cases for that phenotype.

### Genotypes

The quality control (QC), phasing, and imputation was performed centrally as previously described^11^. In brief, the release included 488,377 samples genotyped on the Applied Biosystems UK BiLEVE Axiom Array (UKBL) or the Applied Biosystems UK Biobank Axiom Array (UKBB). The pre-imputation QC included removing markers with large genotype frequency differences due to batch, plate, array, or sex based on Fisher’s exact tests; departures from Hardy–Weinberg equilibrium tested in Fisher’s exact test; discordance across control replicates; and, an overall missing rate > 5% or an overall minor allele frequency (MAF) < 0.0001. Further, samples that were identified as heterozygosity or missingness outliers were excluded. After applying the QC filters, a total of 487,442 samples and 670,739 autosomal markers were phased with SHAPEIT3 and imputed with IMPUTE4 using a combination of the Haplotype Reference Consortium (HRC) 1.1, UK10K, and the 1000 Genomes Phase 3 reference panel. Imputed genotypes from the UK Biobank March 2018 release were used. Unrelated participants of self-reported white British ancestry and European ethnicity based on principal component analysis who passed the genotype QC and had not withdrawn consent at the time of analyses were included (n = 337,484). We included up to 16.5 M genetic markers with a minor allele count ≥ 20 in cases and controls, and a MaCH r^2^ ≥ 0.8. Eighteen phenotypes with ≥ 200 cases in the cohort remained and were tested in the GWAS analyses.

### Association analyses

In total, 81,179 (24%) of the participants had at least one diagnosis indicating a bacterial or viral infection. The number of cases (before or after UK Biobank baseline) for each phenotype included in the GWAS is listed in Table 1. Assuming an additive model, we tested the association between the genotype dosages of each marker and the infection phenotype using logistic regression (Firth’s penalized logistic regression in case of non-convergence) using PLINK2^12^. A linear regression with age, sex, PC1-40, and genotype batch (three levels including UKBL interim release, UKBB interim release, and UKBB second release) as predictors and all infections as the outcome was performed in all individuals included in the GWAS. From this model, all PCs up to PC23 reached *P* < 0.001 and were included as covariates in the GWAS. Associations with *P* values < 5e−8 were considered significant.

**Table 1:** ICD-10 codes and the number of cases and controls per phenotype included in the GWAS.

Phenotypes	Cases	Controls
**Abdominal infections**	34,948	302,536
D73.3, K35–37, K57, K61, K63.0, K65, K75.0, K81, K83.0		
**Respiratory tract infections**	23,560	313,924
Bacterial pneumonia A48.1, A70, J13, J14, J15, J16, J18		
Influenza and viral pneumonia J09–J12		
Other A15, A16, A20.2, A21.2, A22.1, A31.0, A36.0, A36.1, A36.2, A37, A38, B01.2, B05.2, B05.3, B25.0, J01–06, J17, J20–22, J32, J36.9, J39.0, J39.1, J40, J44.0, J44.1, J85.1, J85.2, J85.3, J86, H66		
**Urinary tract infections (UTI)**	13,754	323,730
Cystitis N30.0, N30.9		
Pyelonephritis N10		
Other N15.1, N39.0, N41.0, N41.3		
**Skin and musculoskeletal infections**	11,243	326,241
Skin infections A36.3, A46, J34.0, L00–L05, L08		
Musculoskeletal infections B33.0, M00, M01.0, M01.1, M01.3, M01.4, M01.5, M46.2, M46.3, M46.5, M49.0, M49.1, M49.2, M60.0, M65.0, M65.1, M71.0, M71.1, M86		
**Bacterial pneumonia**	11,133	326,351
A48.1, A70, J13, J14, J15, J16, J18		
**Skin infections**	10,427	327,057
A36.3, A46, J34.0, L00–L05, L08		
**Gastroenteritis**	10,154	327,330
Bacterial gastroenteritis A00–A04		
Viral gastroenteritis A08		
Other A09		
**Sepsis**	4840	332,644
A39.2, A40, A41		
**Specified viral infections**	3759	333,725
A90–99, B00–06, B08, B09, B15–19, B20–27, B30, B33, B34, B97		
**Bacterial gastroenteritis**	2506	334,978
A00–A04		
**Urogenital (non-UTI) infections**	2141	335,343
N34.0, N41.1, N41.2, N43.1, N45, N76.0, N76.2, N76.4		
**Cystitis**	1457	336,027
N30.0, N30.9		
**Musculoskeletal infections**	1189	336,295
B33.0, M00, M01.0, M01.1, M01.3, M01.4, M01.5, M46.2, M46.3, M46.5, M49.0, M49.1, M49.2, M60.0, M65.0, M65.1, M71.0, M71.1, M86		
**Viral gastroenteritis**	899	336,585
A08		
**Central nervous system infections**	750	336,734
A17, A20.3, A32.1, A39.0, A80–89, B00.3, B00.4, B01.0, B01.1, B02.0, B02.1, B02.2, B05.0, B05.1, B06.0, B26.1, B26.2, G00, G01, G02.0, G04–07		
**Heart infections**	704	336,780
A39.5, I30.1, I32.0, I33, I38, B33.2, I40.0, I41.0, I41.1		
**Influenza and viral pneumonia**	479	337,005
J09–J12		
**Sexually transmitted diseases**	222	337,262
A50–58, A60, A63–64		

Only phenotypes with ≥ 200 cases were included in the analysis.

A full description of all phenotypes is provided in Supplementary Table S1.

We identified regions containing one or more genome-wide significant SNPs by screening a window of 500 kb adjacent to the first genome-wide significant SNP on each chromosome sorted by genomic position. If no additional SNPs were identified, the region was limited to that specific SNP, and screening was continued at the next GWAS-significant SNP. If additional GWAS-significant SNPs were found, the window was expanded with 300 kb from the last SNP, and screened for additional GWAS-significant SNPs, until there were no more such SNPs within the next 300 kb. Within each region, the SNP with lowest *P* value was assigned as the index SNP. For each region, conditional association analysis was performed adjusting for all index SNPs found on the chromosome. This distance-based pruning followed by conditional analysis was repeated until no SNPs reached *P* value < 5e−8. Significant, independent loci with MAF ≥ 1% discovered in the GWAS were compared across the infection phenotypes in this study and all ICD categories (e.g., K57, J18) with ≥ 200 cases and other relevant phenotypes (e.g., smoking, body mass index [BMI]) within the UK Biobank cohort. Linear or logistic regression adjusting for the same covariates as in the infection GWAS was applied for 39 SNPs vs. 743 phenotypes yielding a Bonferroni corrected threshold for significance of *P* = 1.7e−6.

### GWAS catalog and LocusZoom plots

Index SNPs across all traits were linkage disequilibrium (LD) pruned, based on r^2^ < 0.1 in 500 kb windows with LD data from European samples from 1000 Genomes phase 3 (v5), creating a list of independent loci for all phenotypes. GWAS catalog data (downloaded 2019-06-26, available at https://www.ebi.ac.uk) within 250 kb of each independent locus was extracted and pairwise r^2^ was calculated between each index SNP and catalog hit. For multi-allelic markers, r^2^ was calculated for the alternate allele with the highest allele frequency. LD calculations were not performed for markers that were not present in 1000G or monomorphic in the European subset. These variants are included in the tables, but with r^2^ set to missing. Distance to the nearest gene was calculated as the distance from the index SNP to the transcript start or end position (whichever was closest). Regional plots of the association test results were generated for significant loci using LocusZoom v1.4^13^ using LD data and GWAS catalog annotations. In the interpretation of result, we focused on SNPs with MAF ≥ 1% and GWAS catalog hits with r^2^ ≥ 0.30 and distance ≤ 100 kb from the genetic variant identified in our study. Other hits or nearby genes located within 250 kb from the index SNP, are sometimes discussed if considered biologically relevant to the infectious phenotype. In these cases, the effect allele frequency (EAF), distance and r^2^ for that specific SNP are specified in the text.

### Fine-mapping of the HLA region

Fine mapping of the human leucocyte antigen (HLA) region was performed due to the critical functions of HLA genes in the immune response, the highly polymorphic nature of the region and high LD between alleles at nearby loci. Imputation of all 11 HLA loci was done centrally using HLA*IMP:02 following the same pre-imputation QC as described for the genome-wide imputation^11^. Dosages for all possible alleles at each HLA locus were tested in separate logistic regression models adjusting for the same covariates as described for the GWAS. Non-tested alleles were assigned a dosage of 0. Only alleles with a minor allele count ≥ 20, calculated separately in cases and controls, were tested. After Bonferroni correction, associations with *P* < 1.6e−5 were considered significant.

### Functional annotation

The Summary data-based Mendelian Randomization (SMR) approach^14^ was applied to determine whether associations between SNP and infection phenotype could be explained by known gene expression. SMR analysis was performed by jointly analysing the infection GWAS results and publicly available expression quantitative trait locus (eQTL) summary statistics, thereby assessing potential functional significance of the identified loci pointing to a causal gene. SMR 1.02 was used with the default settings including GWAS results with MAF ≥ 1%. To assess if the GWAS and eQTL association with the phenotype was due to a single shared genetic variant rather than multiple variants in LD with independent effects on the phenotype the heterogeneity in dependent instruments (HEIDI) test was applied^14^. Gene expression data from eQTL studies were obtained from the Genotype Tissue Expression project (GTEx) V7 release^15^ and LD data from 1000G phase 3 (v5) EUR was used. To limit the number of tests only gene expression in biologically plausible tissues were considered. Gene expression in the spleen and whole blood was considered potentially important for the immune defence and therefore relevant for all phenotypes. Also, specific tissues were selected for phenotypes where significant SNPs had been found in the GWAS (Table S2).

### Heritability

Narrow-sense heritability (h^2^) explained by additive SNP effects was calculated using LDSC v.1.0.0^16^ and observed scale heritability estimates are reported. Infection phenotypes with at least 5000 cases (corresponding to an effective sample size of ~ 20,000) were included using a subset of the SNPs with likely high imputation quality passing the quality filters described for the GWAS, MAF ≥ 1%, and inclusion in HapMap3. For comparison, we also estimated the SNP heritability by Haseman-Elston (HE) regression in GCTA 1.92.1^17^. Directly genotyped SNPs with MAF ≥ 1% were used to construct the genetic relationship matrix. Results from the HE regression based on the cross-product with the standard error computed using the Jackknife approach are reported.

All methods were carried out in accordance with relevant guidelines and regulations.

## Results

### Genotype–phenotype associations

**Figure 1:**
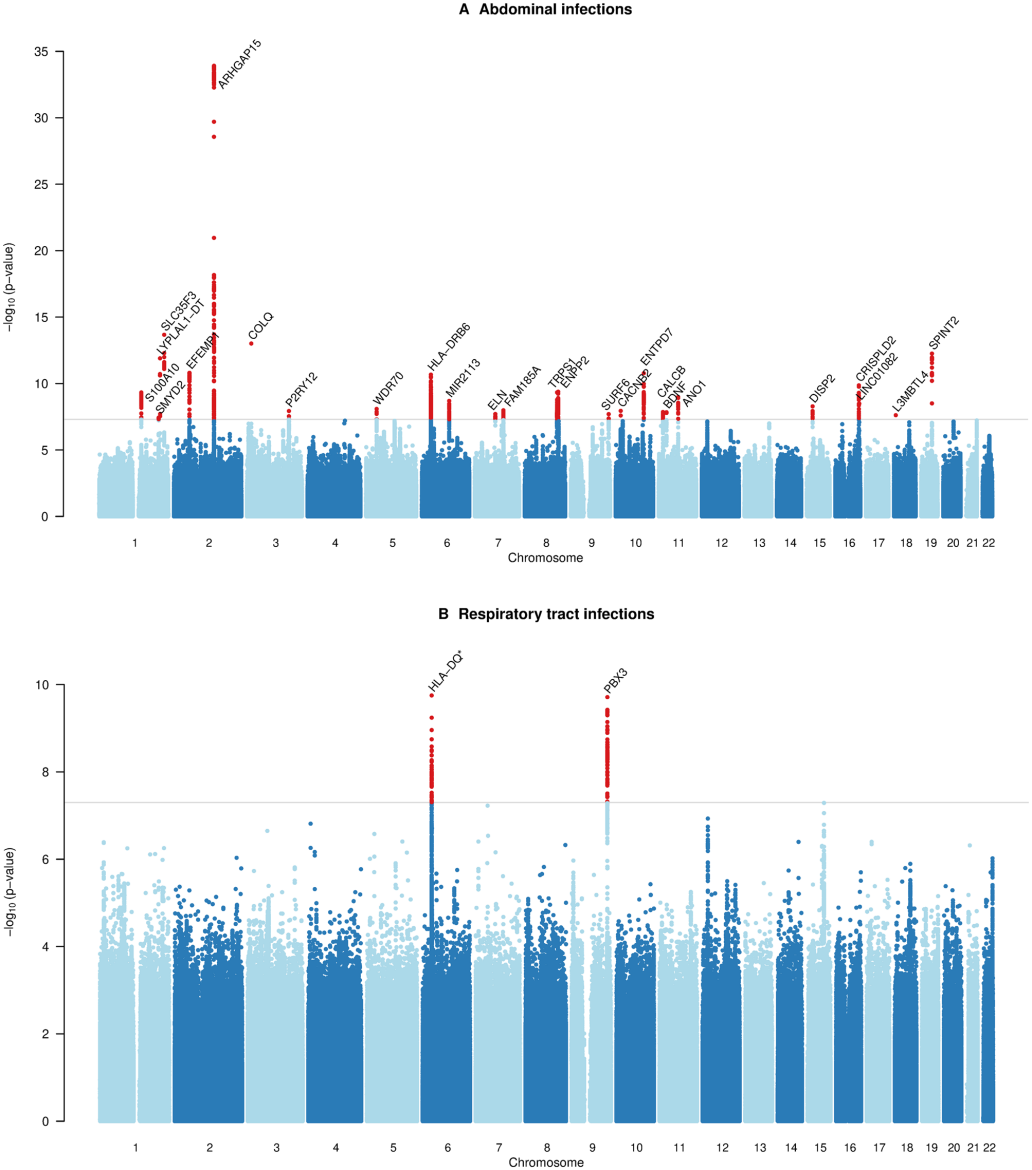
Manhattan plots showing associations of genetic variants with (**A**) abdominal infections and (**B**) respiratory tract infections in the UK Biobank cohort (n = 337,536). The asterisk * indicates that associations were found for multiple genes belonging to the HLA-DQ group (e.g., HLA-DQA1, HLA-DQB1). Negative log_10_-transformed *P* values for each SNP (y axis) are plotted by chromosomal position (x axis). The grey line represents the threshold for genome-wide statistically significant associations (*P* = 5e−08). Red points represent significant hits, and each significant locus is annotated with the nearest gene.

**Figure 2:**
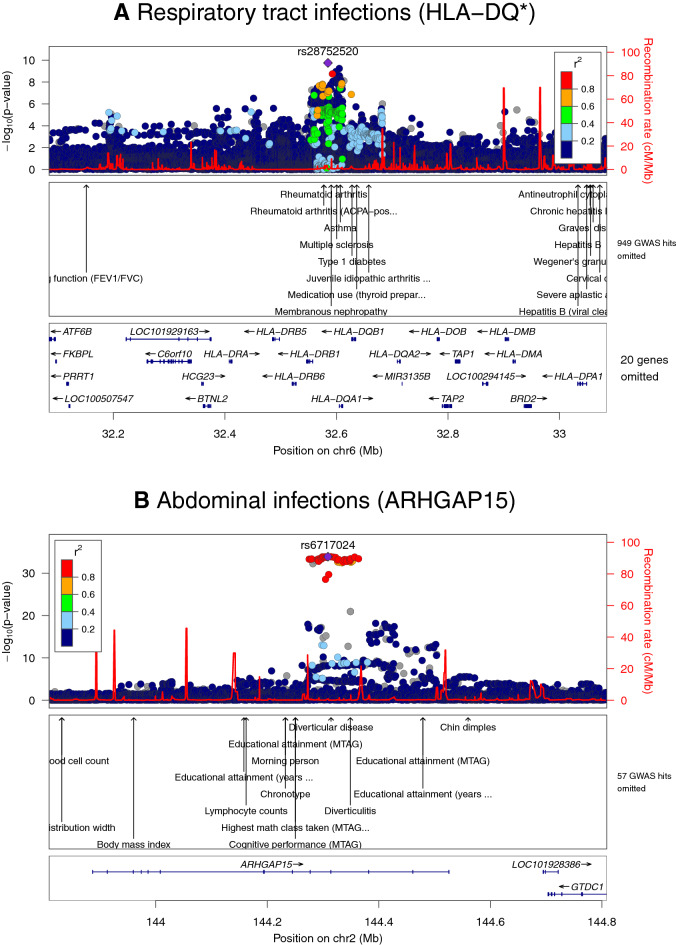
Regional association and linkage disequilibrium plots for the (**A**) HLA-DQ* locus in relation to respiratory tract infections and the (**B**) *ARHGAP15* locus in relation to abdominal infections. The y axis represents the negative log_10_ of the variant *P* values, and the x axis represents the position on the chromosome, with the name and location of genes shown in the bottom panel. The SNP with the lowest *P* value in the region is marked by a purple diamond. The colours of the other SNPs indicate the correlations of these SNPs with the lead SNP. Plots were generated with LocusZoom.

In total, 57 unique genome-wide significant loci were found across all phenotypes (Table 2, Fig. S1). Neither the QQ plots (Fig. S2) nor the genomic control lambda metrics (Table S3) indicated a major inflation of the association test statistics for any of the phenotypes. We detected significant variants in the HLA region on chromosome 6 (nearby genes; *HLA-DQA1, HLA-DQB1, HLA-DQB1-AS1, HLA-DRB1, HLA-DRB5* and *HLA-DRB6*) for abdominal infections and RTIs (Figs. 1, 2A). Fine mapping of the HLA region revealed significant associations with HLA-DQA1, HLA-DRB1, and HLA-DRB4 locus alleles (Table S4). HLA-DQ and HLA-DR are major histocompatibility complex (MHC) class II molecules that play a key role in the adaptive immune response, especially against bacterial infections, by presenting pathogen antigens mainly to the CD4 + T helper cells^18^.

**Table 2:** Significant (*P* < 5e−08) SNPs and genetic loci associated with bacterial and viral infections in the GWAS.

Phenotype	SNP	CH	Position (b37)	Effect allele	Other allele	EAF cases	EAF controls	Odds ratio (95% CI)	Beta	SE	P	MACH-Rsq	Nearby genes (+ / − 100 kb)
Abdominal infections	rs6717024	2	144,308,780	C	G	0.2	0.18	1.133 (1.111–1.156)	0.125	0.010	1.22e−34	1.00	ARHGAP15
	rs4333882	1	234,352,899	G	A	0.2	0.19	1.081 (1.060–1.103)	0.078	0.010	2.14e−14	0.97	MIR4671, SLC35F3
	rs7609897	3	15,502,681	T	G	0.2	0.21	0.925 (0.907–0.943)	− 0.078	0.010	9.76e−14	0.91	COLQ, EAF1, HACL1, METTL6, MIR4270
	rs760364725	19	38,757,315	CA	C	0.49	0.48	1.062 (1.045–1.079)	0.060	0.008	5.59e−13	0.94	C19orf33, CATSPERG, DPF1, KCNK6, PPP1R14A, SIPA1L3, SPINT2, YIF1B
	rs61823192	1	219,294,570	T	C	0.026	0.03	0.828 (0.785–0.873)	− 0.189	0.027	1.26e−12	0.90	LYPLAL1, LYPLAL1-DT
	rs1802575	2	56,093,204	C	G	0.14	0.13	1.081 (1.056–1.107)	0.078	0.012	1.56e−11	1.00	EFEMP1, MIR217HG
	rs11428277	10	101,425,013	G	GA	0.19	0.18	1.075 (1.052–1.098)	0.072	0.011	1.69e−11	0.91	COX15, CUTC, ENTPD7, SLC25A28
	rs570640158	6	32,517,793	C	T	0.27	0.26	1.070 (1.050–1.092)	0.068	0.010	2.13e−11	0.81	HLA-DQA1, HLA-DRB1, HLA-DRB5, HLA-DRB6
	rs4782673	16	84,884,072	T	G	0.28	0.3	0.945 (0.928–0.961)	− 0.057	0.009	1.37e−10	1.00	CRISPLD2, USP10
	rs7464710	8	120,624,386	G	C	0.25	0.26	0.943 (0.926–0.959)	− 0.059	0.009	4.13e−10	0.98	ENPP2
	rs2049865	8	116,588,546	C	A	0.41	0.42	0.950 (0.935–0.965)	− 0.051	0.008	4.67e−10	0.98	TRPS1
	rs61817723	1	151,920,865	A	G	0.27	0.28	0.945 (0.928–0.961)	− 0.057	0.009	4.71e−10	0.99	C2CD4D-AS1, NBPF18P, S100A10, S100A11, THEM4, THEM5
	rs2276068	11	70,007,484	C	G	0.49	0.48	1.050 (1.034–1.067)	0.049	0.008	1.04e−09	1.00	ANO1, FADD, LINC02584, LOC101928443
	rs9372625	6	98,344,031	A	G	0.37	0.38	0.951 (0.936–0.966)	− 0.050	0.008	2.02e−09	0.99	
	rs2280028	16	86,233,413	A	G	0.13	0.14	0.932 (0.911–0.955)	− 0.070	0.012	3.23e−09	0.99	LINC01081, LINC01082, LINC02135
	rs71472433	15	40,649,609	C	A	0.17	0.17	1.064 (1.041–1.087)	0.062	0.011	5.24e−09	0.99	ANKRD63, BAHD1, BUB1B-PAK6, CCDC9B, DISP2, INAFM2, IVD, KNSTRN, PAK6, PHGR1, PLCB2
	rs2973068	5	37,778,273	G	C	0.24	0.25	0.947 (0.931–0.964)	− 0.054	0.009	8.15e−09	0.99	GDNF, GDNF-AS1, WDR70
	rs761264338	7	102,444,156	GGAAG	G	0.34	0.33	1.050 (1.032–1.069)	0.049	0.009	1.03e−08	0.98	FAM185A, FBXL13, RASA4DP
	rs1888693	10	18,440,444	A	G	0.33	0.34	0.952 (0.936–0.969)	− 0.049	0.009	1.14e−08	1.00	CACNB2
	rs3732760	3	151,074,941	C	A	0.38	0.37	1.048 (1.032–1.065)	0.047	0.008	1.17e−08	1.00	GPR87, IGSF10, MED12L, P2RY12, P2RY13, P2RY14
	rs575909118	11	15,065,235	CT	C	0.27	0.27	1.053 (1.035–1.072)	0.052	0.009	1.40e−08	0.98	CALCA, CALCB, INSC
	rs11030119	11	27,728,102	A	G	0.3	0.31	0.951 (0.935–0.968)	− 0.050	0.009	1.44e−08	1.00	BDNF, BDNF-AS, LINC00678
	rs60731259	7	73,439,465	CA	C	0.066	0.061	1.096 (1.063–1.131)	0.092	0.016	1.96e−08	0.99	ELN, LIMK1
	rs9411377	9	136,145,404	A	C	0.28	0.29	0.950 (0.934–0.967)	− 0.051	0.009	2.01e−08	0.96	MED22, OBP2B, RPL7A, SNORD24, SNORD36A, SNORD36B, SNORD36C, STKLD1, SURF1, SURF2, SURF4, SURF6
	rs377411728	18	6,305,754	T	C	0.00071	0.00031	2.641 (1.878–3.714)	0.971	0.174	2.42e−08	0.83	L3MBTL4, L3MBTL4-AS1, MIR4317
	Not available	1	214,458,533	C	CT	0.46	0.45	1.046 (1.030–1.063)	0.045	0.008	3.67e−08	0.99	PTPN14, SMYD2
Gastroenteritis	rs115809651	4	162,414,623	A	G	0.0014	0.00046	3.525 (2.359–5.269)	1.260	0.205	8.07e−10	0.86	FSTL5
	rs138491114	22	47,432,980	A	G	0.0035	0.0018	1.986 (1.557–2.532)	0.686	0.124	3.14e−08	0.97	TBC1D22A
	rs772878892	16	49,404,415	G	A	0.0011	0.00032	3.597 (2.274–5.690)	1.280	0.234	4.77e−08	0.90	C16orf78, CBLN1
a. Bacterial gastroenteritis	rs143977447	5	164,646,152	A	C	0.011	0.0053	2.375 (1.787–3.156)	0.865	0.145	2.20e−09	0.85	
	rs547484470	7	68,931,296	A	C	0.0045	0.0014	3.710 (2.396–5.744)	1.311	0.223	4.13e−09	0.88	
b. Viral gastroenteritis	rs116879283	7	52,434,143	C	T	0.017	0.0063	2.956 (2.033–4.299)	1.084	0.191	1.49e−08	0.90	
Heart infections	rs564399474	13	54,865,197	G	A	0.016	0.0039	4.527 (2.930–6.994)	1.510	0.222	1.02e−11	0.93	MIR1297
	rs182592259	14	96,788,318	A	T	0.016	0.005	3.589 (2.323–5.546)	1.278	0.222	8.73e−09	0.88	AK7, ATG2B, BDKRB1, BDKRB2, GSKIP
	rs139809494	17	79,796,214	T	C	0.019	0.0064	3.139 (2.117–4.655)	1.144	0.201	1.30e−08	0.96	ALYREF, ANAPC11, ARHGDIA, GCGR, MAFG, MAFG-DT, MCRIP1, NPB, P4HB, PCYT2, PPP1R27, PYCR1, SIRT7
	rs2181386	14	94,039,907	G	A	0.16	0.12	1.490 (1.294–1.716)	0.399	0.072	3.51e−08	1.00	UNC79
Respiratory tract infections	rs28752520	6	32,584,739	C	T	0.24	0.25	0.931 (0.911–0.951)	− 0.072	0.011	1.77e−10	0.99	HLA-DQA1, HLA-DQB1, HLA-DQB1-AS1, HLA-DRB1, HLA-DRB5, HLA-DRB6
	Not available	9	128,648,077	ATG	A	0.34	0.35	0.937 (0.919–0.956)	− 0.065	0.010	1.94e−10	0.98	PBX3
a. Bacterial pneumonia	rs374402947	16	89,154,513	T	C	0.0014	0.00048	3.391 (2.300–4.998)	1.221	0.198	6.86e−10	0.88	ACSF3, CDH15, LINC00304, LINC02138
	rs77438700	15	78,906,637	G	A	0.064	0.074	0.839 (0.795–0.887)	− 0.175	0.028	7.72e−10	0.97	CHRNA3, CHRNA5, CHRNB4, HYKK, PSMA4
	rs532298826	4	138,378,079	A	T	0.0016	0.00064	3.056 (2.093–4.461)	1.117	0.193	6.62e−09	0.80	LINC02172, PCDH18
	rs763947133	5	127,719,333	T	C	0.00098	0.00029	3.971 (2.466–6.393)	1.379	0.243	1.33e−08	0.81	FBN2
b. Influenza and viral pneumonia	rs376768393	3	154,969,551	C	T	0.021	0.0064	3.850 (2.434–6.090)	1.348	0.234	8.57e−09	0.87	DWORF, LINC01487, MME
Sepsis	rs532825748	1	169,752,583	G	A	0.0078	0.0041	2.018 (1.586–2.568)	0.702	0.123	1.13e−08	0.89	C1orf112, METTL18, SCYL3, SELE, SELL
	rs147389769	2	231,527,107	T	C	0.007	0.0036	2.130 (1.638–2.769)	0.756	0.134	1.60e−08	0.83	CAB39, LINC01907
	rs564716204	4	151,664,401	T	C	0.0027	0.00092	3.353 (2.200–5.111)	1.210	0.215	1.90e−08	0.82	LRBA
	rs539059490	6	122,025,326	G	T	0.0073	0.0039	2.006 (1.567–2.568)	0.696	0.126	3.65e−08	0.89	
Sexually transmitted diseases	rs62441491	7	41,892,859	A	G	0.056	0.018	3.438 (2.274–5.199)	1.235	0.211	4.73e−09	0.94	INHBA-AS1
Skin infections	rs6595799	5	127,408,683	C	T	0.28	0.27	1.092 (1.058–1.127)	0.088	0.016	2.39e−08	0.98	LINC01184, SLC12A2
Specified viral infections	rs568156598	7	94,453,230	G	A	0.0029	0.00091	3.747 (2.397–5.858)	1.321	0.228	6.68e−09	0.85	PPP1R9A
Urinary tract infections (UTI)	rs771331833	8	118,513,358	A	C	0.0013	0.00049	2.989 (2.096–4.262)	1.095	0.181	1.62e−09	0.93	MED30
	Not available	3	47,875,364	A	AT	0.009	0.0064	1.471 (1.285–1.684)	0.386	0.069	2.81e−08	0.90	DHX30, MAP4, MIR1226, SMARCC1
	rs189319388	3	7,867,797	T	C	0.0077	0.0055	1.527 (1.313–1.775)	0.423	0.077	4.63e−08	0.81	GRM7
a. Cystitis	rs192629083	7	14,037,179	T	C	0.0077	0.0025	3.702 (2.396–5.721)	1.309	0.222	3.50e−09	0.86	ETV1
	rs77261774	4	60,641,124	A	G	0.034	0.02	1.788 (1.461–2.188)	0.581	0.103	1.49e−08	1.00	LINC02429
Urogenital (non-UTI) infections	rs188692128	8	126,584,263	T	C	0.005	0.0017	3.589 (2.282–5.645)	1.278	0.231	3.13e−08	0.85	
	Not available	1	240,891,604	T	TA	0.0052	0.0018	3.456 (2.219–5.381)	1.240	0.226	4.30e−08	0.83	RGS7

Phenotypes are listed in alphabetical order.

#### Abdominal infections

Twenty-six significant genetic variants were associated with abdominal infections (Table 2, Fig. 1A). The results for this phenotype were largely driven by ICD-10 code K57; intestinal diverticular disease and diverticulitis. A sensitivity analysis was performed where K57 was removed from the case definition of abdominal infections. With this updated definition of abdominal infections only three loci reached nominal significance (lead variants rs11428277, rs2049865, and rs377411728), while almost all loci reached *P* value < 5e−8 when tested for an association with K57 alone (data not shown). One locus (lead variant rs377411728) reached *P* value < 1e−5 for both K57 and the abdominal infection phenotype excluding K57.

The strongest hit was an intronic variant of the *ARHGAP15* gene (chr2:rs6717024, *P* = 1.22e−34) (Fig. 2B). In an in vivo sepsis model, lack of ArhGAP15 (Rho GTPase-activating protein 15), which functions as a negative regulator of multiple neutrophil functions, induced cellular elongation but resulted in more efficient neutrophil migration, phagocytosis, and bacterial killing^19^. Based on these data, ARHGAP was suggested as a therapeutic target to enhance the antibacterial activity of white blood cells and decrease systemic inflammation in septic patients. *CRISPLD2* (lead variant rs4782673, *P* = 1.37e−10), which is expressed in multiple tissues and leukocytes, has previously been associated with mortality in sepsis^20^. In a small case–control study, CRISPLD2 was reduced in patients with septic shock and showed a negative correlation with the bacterial infection biomarker procalcitonin^21^. In mice, administration of recombinant CRISPLD2 was protective for endotoxin shock, presumably through inhibition of the binding of bacterial lipopolysaccharide (LPS) endotoxins to target cells, and consequently reduced induction of the TNF-a and IL-6 cytokine production^22^.

Variants in the *ARHGAP15* locus were associated with diverticular disease in a GWAS using the UK Biobank cohort^23^ and in cohort of Icelandic and Danish cases and controls^24^. In addition to *ARHGAP15,* most other variants, in or nearby genes *SLC35F3*, *CALCB*, *COLQ*, *EFEMP1*, *LYPLAL1-DT*, *CRISPLD2*, *TRPS1*, *S100A10, ANO1, LINC01082, DISP2, CACNB2*, *BDNF*, *P2RY14*, *WDR70*, *ELN*, *FAM185A*, *ENPP2*, *ENTPD7*, *ABO* (close to *SURF6*), *PPP1R14A* (close to *SPINT2*) and *MIR2113*, were also located closely to SNPs previously associated with diverticular disease^23^. The protein encoded by *COLQ* (lead variant rs7609897, *P* = 9.76e−14) influences smooth muscle motility and the neuromuscular junctions between nerve cells and muscle cells, suggesting a biological function in the development of intestinal diverticula. Variants in the *COX15* locus (lead intronic variant, chr10:rs11428277; *P* = 1.69e−11) were previously associated with colorectal cancer^25^ and Crohn’s disease^26^. The COX15 protein is localized in the inner mitochondrial membrane and has a key function in the electron transport chain. Bacterial invasion in the intestinal mucosa secondary to inflammation or cancer is a plausible biological explanation for the observed associations.

Moreover, several genetic variants were located nearby SNPs or genes of potential importance to susceptibility to other types of infections, the host immune defence and other intra-abdominal conditions. A SNP in the *EFEMP1* locus (lead variant rs1802575, 3’UTR, *P* = 1.56e−11), was previously associated with a history of childhood ear infection^8^. Decreased expression of *EFEMP1* (epidermal growth factor-containing fibulin-like extracellular matrix protein) in hepatocellular cancer cells is a predictor of tumour spread and metastasis, and consequently worse prognosis^27^. Interestingly, EFEMP1 acts by promoting SEMA3B, which belongs to the semaphorin family of proteins that regulate multiple physiologic processes including the immune response and cell migration. Reduced levels of SEMA3B in fibroblast-like synoviocytes was found in patients with rheumatoid arthritis, suggesting a role also in the development of autoinflammatory disease^28^. Variants in the *SLC35F3* locus (lead variant, chr1:rs4333882; *P* = 2.14e−14) have been reported associated with levels of the pro-inflammatory cytokine IL-6^29^. The biological function of *SLC35F3* is unknown, but IL-6 has a key role in the acute phase response to infections by stimulating the production of neutrophils. SNPs in or nearby *TRPS1* (lead variant, rs2049865, *P* = 4.67e−10) were previously associated with white blood cells and cytokines^30^, and MIR2113 (distance 128 kb from rs9372625, *P* = 2.02e−9) with the composition of the gut microbiota^31^.

#### Respiratory tract infections

Seven independent loci were associated with RTI phenotypes (all RTIs, n = 2; bacterial pneumonia, n = 4; influenza and viral pneumonia, n = 1) (Table 2, Fig. 1B). The strongest hit associated with the combined phenotype of all RTIs (chr6:rs28752520, *P* = 1.77e−10) (Fig. 2A), located in the *HLA-DQA1* locus, was previously found to be associated with blood protein levels^32^. Other variants close to our index SNP, but not strongly correlated, have also shown associations with common infections; plantar warts (distance = 0 kb, r^2^ = 0.47 according to the GWAS catalog database), childhood ear infection (18 kb, r^2^ = 0.021) and scarlet fever (43 kb, r^2^ = 0.026)^8^. A significant variant on chromosome 9 (position 128,648,077, *P* = 1.94e−10), previously reported to be associated with sleep duration^33^, is located near *PBX3*; variants in this locus have shown association with squamous cell lung carcinoma^34^. The gene product, pre-B-cell leukaemia transcription factor 3, induced inflammatory response in sepsis in a murine infection model by acting as a competing endogenous RNA for HMGB1 (high-mobility group protein 1)^35^. HMGB1 is produced by macrophages in response to bacterial infections, functioning as an endotoxin-induced cytokine mediator of inflammation, and has been proposed a potential therapeutic target for sepsis^36^.

Previous studies of schizophrenia^37^, cigarette smoking, chronic pulmonary disease^38^ and lung cancer^39^ reported associations with SNPs that were adjacent (< 10 kb), but not strongly correlated (r^2^ ≤ 0.246), to one of the SNPs associated with bacterial pneumonia (lead variant, rs77438700, *P* = 7.72e−10). Nearby genes of interest include *CHRNA3* and *CHRNA5*; variants in this locus are associated with chronic obstructive pulmonary disease and lung cancer^40^. CHRNA3 and CHRNA5 encode the alpha-type subunit of a cholinergic receptor, which likely mediates the effects of nicotine on the brain. Due to their association with nicotine dependence, the causal variants at this locus probably serve as a determinant of smoking behaviour, subsequently increasing the risks of chronic lung disease and bacterial pneumonia.

#### Sepsis

The GWAS revealed only four rare variants associated with sepsis (Table 2) and no significant correlations were found in the GWAS catalog database. Our findings should be interpreted with caution due to the low frequency and limited sample size of this phenotype (n = 4840). Nearby genes of interest include *SELE* and *SELL*, which encode the leukocyte cell adhesion receptors Selectin E and L that are involved in leukocyte/endothelium interactions during interleukin-induced inflammation. SELE is associated with Leukocyte Adhesion Deficiency (LAD), a rare autosomal recessive disorder typically presenting with recurrent severe bacterial infections^41^. Selectin L facilitates entry of lymphocytes into the extracellular space^42^, which is an integral process in the immune response to sepsis. Another associated rare variant (EAF cases = 0.0027, lead SNP chr4:rs564716204, *P* = 1.90e−08) was located nearby *LRBA*. LRBA (LPS responsive beige-like anchor protein) deficiency is an autosomal recessive genetic disorder caused by mutations resulting in reduced expression and function of the cytotoxic T lymphocyte-associated protein 4 (CTLA4)^43^. This condition is associated with low levels of immunoglobulins (IgG, IgM, IgA), repeated infections due to impaired humoral immune response, and increased risk of autoinflammatory diseases (e.g., diabetes mellitus, inflammatory bowel disease).

#### Other phenotypes

Multiple loci, most of which are novel in the context of infectious diseases, were found for the remaining phenotypes: gastroenteritis (n = 6), heart infections (n = 4), sexually transmitted diseases (n = 1), skin infections (n = 1), specified viral infections (n = 1), UTI (n = 5) and urogenital (non-UTI) infections (n = 2) (Table 2). A genetic variant associated with skin infections in our study (lead SNP chr5:rs6595799, *P* = 2.39e−08) is highly correlated (r^2^ ≥ 0.9) and close to SNPs near *LINC01184*. LINC01184 is a long intergenic non-protein coding RNA that is differentially expressed in many types of cancers that has previously been reported associated with cancer^44,45^, blood cell traits^46^ and other phenotypes^47^. One of the strongest hits for heart infections in our study (chr14:rs182592259, *P* = 8.73e−09) was located near *BDKRB2*, which encodes the bradykinin B2 receptor that has a protective role in the development of hypertension and cardiovascular disease^48^, thereby potentially affecting also the vulnerability to infections.

### Functional annotation

**Figure 3:**
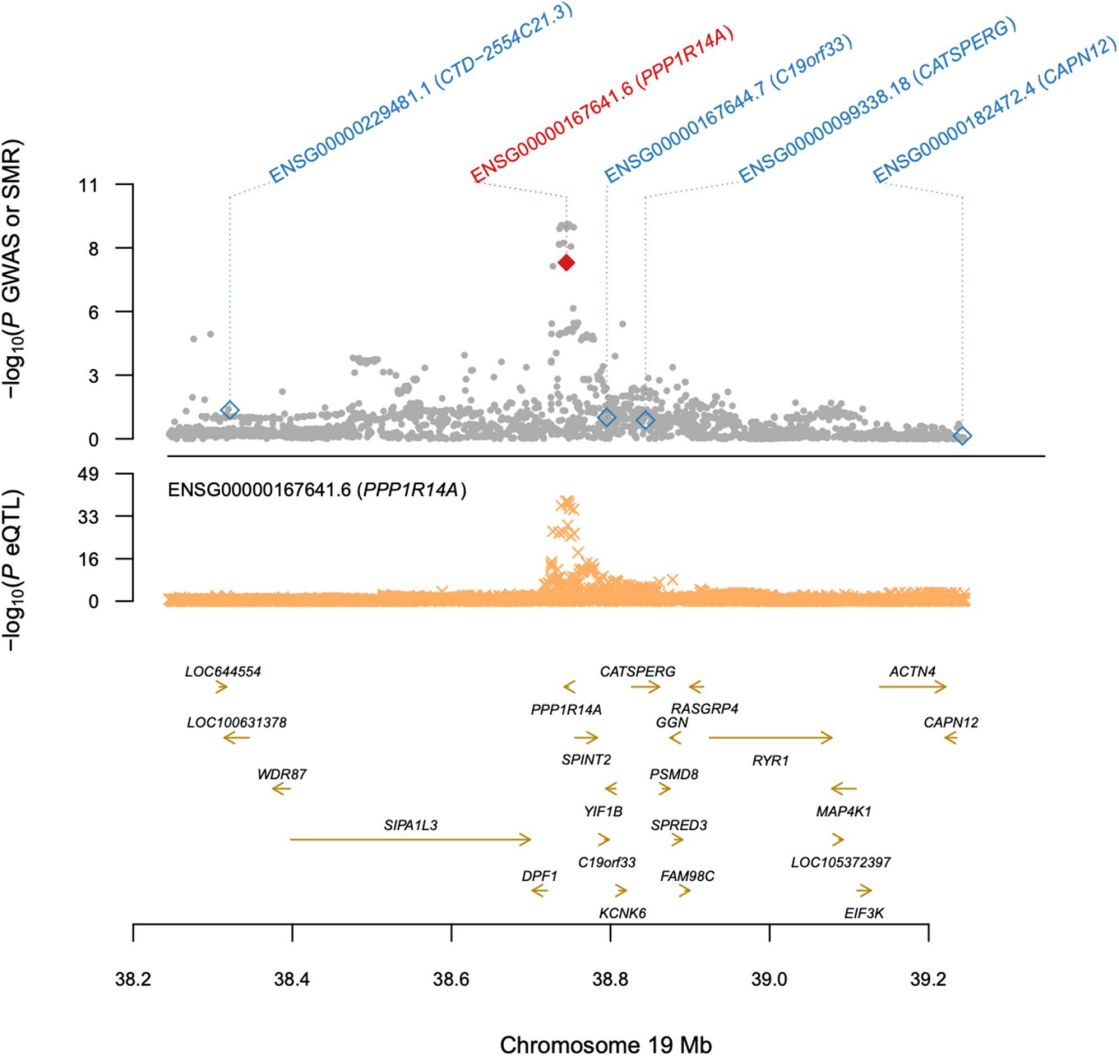
eQTL colocalization between GWAS signal for abdominal infections and *PPP1R14A* expression in the colon. Neighbouring genes do not show the same colocalization.

We identified a total of 91 colocalization events representing 4, 15 and 23 unique traits, tissues and genes respectively. *PPP1R14A* showed the strongest colocalization with abdominal infections, and colocalized with eQTL signals in both sigmoid and transverse colon tissue with lead variants in strong LD (r^2^ > 0.98; strongest association lead SNP rs4803934, SMR, *P* = 4.68e−10) (Table S5). Neighboring genes did not show a similar pattern of colocalization with the GWAS signal in this locus (Fig. 3). PPP1R14A, also known as CPI-17, belongs to the protein phosphatase 1 (PP1) inhibitor family which has a key role in the adjustment of smooth muscle contraction in response to physiological stimuli^49^. *PPP1R14A* has shown associations with different cancer types in prior large-scale GWASs, including colon and prostate cancer^50,51^. Evidence from these studies points to a transcriptionally mediated effect; imputed *PPP1R14A* expression, derived as a linear combination of cis genotypes associated with expression of the gene, showed association with prostate cancer in two independent cohorts^51^. This locus also shows association with diverticular disease^23^.

We observed that several colocalization events for abdominal disease occurred with genes in the HLA region; SMR and HEIDI analyses identified *HLA-DQA2, HLA-DRB6* and *HLA-DRB1*. Due to the complex LD structure in the HLA region, there is likely to be additional pleiotropy occurring with these discoveries. Although we performed HLA region fine-mapping to detect more closely resolved association signals, we did not perform SMR and HEIDI analyses with the fine-mapped data. Colocalizations for HLA regions were observed in 11 distinct tissues including whole blood and spleen; many tissues likely share eQTLs that underlie these results. We also observed a colocalization between *ABO* expression and abdominal infections in the adipose visceral omentum (lead SNP rs505922; *P* = 6.91e−06). Associations between blood types and infections were amongst the earlier identified associations between molecular traits and phenotypes^52^. There is evidence that individuals with different blood types have varying levels of susceptibility to acute pyelonephritis^53^, presumably mediated by the expression of receptors in the endothelium, and abdominal infections^54^. Finally, we observed colocalizations between abdominal infections and the colon expression of *NOV* (also called *CCN3*, lead variant rs61100635, *P* = 1.75e06) and *DISP2* (rs2289328; *P* = 1.27e−06).

### Heritability

The narrow-sense heritability on the observed scale was low (0–4%) for all phenotypes (Table S6) with the highest heritability found for abdominal infections. The difference in heritability could partly be related to the phenotype definitions; the phenotype of abdominal infections was more homogenous than RTIs, which included multiple infections of varying severity and pathogens. Moreover, the genetic component is likely higher in endogenous infections, such as abdominal infections, which are normally caused by bacteria of the host’s microbiome, compared to exogenous infections, including viral RTIs or gastroenteritis, which depend on exposure and acquisition of a transmittable pathogen.

### Phenome-wide associations in the UK Biobank

The analysis of shared significant loci across infectious and non-infectious phenotypes in the UK Biobank cohort revealed associations between one of the SNPs identified for abdominal infections (rs570640158) located in the HLA region, and phenotypes related to infection, inflammatory and autoimmune diseases, including CRP, asthma (ICD-10 code J45), diabetes mellitus (E10, E11) and rheumatoid arthritis (M05, M06) (Fig. S3B). There were multiple shared SNP associations between abdominal infections and diverticular disease (ICD-10 code K57), as discussed above, and rs77438700 was associated both with bacterial pneumonia (ICD-10 code J18) and smoking.

## Discussion

In this study, we explored genetic determinants of the susceptibility to phenotypes representing 18 bacterial and viral infection entities and identified 57 unique loci associated with at least one of the phenotypes. While many of detected significant variants are novel in the context of infectious diseases, the same or strongly correlated SNPs, and nearby genes of potential relevance in the pathophysiology of infections, were frequently found in previous literature. Most SNPs detected for abdominal infections were located close to loci reported associated to diverticular disease and diverticulitis (ICD-10 code K57), which was also the main driver of results for this phenotype in our study, in a GWAS by Maguire et al.^23^.

As expected, some of the identified loci are associated with infectious diseases or components of the host immune defence against bacterial and viral infections, such as the HLA region. Our findings align with a previous GWAS in which genetic variants in the HLA region were associated with several self-reported infections (e.g., mononucleosis, mumps, pneumonia, and tuberculosis)^8^. Bacterial infections are typically associated with MHC-II genes, and viral infections with the MHC-I region, which is important for peptide recognition in CD8 + cytotoxic T cells. The HLA region is also associated with multiple immunological traits including selective IgA deficiency, the most common primary immunodeficiency in Europeans^55^ and autoimmune diseases such as rheumatoid arthritis^56^, systemic lupus erythematosus^57^ and ulcerative colitis^58^. Interestingly, one study showed that one of the genes and its products, *HLA-DQA2*, is often transferred from cancerous cells to normal cells via extracellular vesicles in malignant colon cancer^59^. The transfer of this and other genes resulted in neoplastic transformation in fibroblasts. Also, alleles in *HLA-DRB1* have shown association with on the composition of the gut microbiome^56^.

Genetic variants in the *TRPS1* (rs2049865, *P* = 4.67e−10) and *LINC01184* (rs6595799, *P* = 2.39e−08) loci, associated with abdominal infections and skin infections, respectively, showed associations with neutrophil and lymphocyte counts in a cohort of ~ 175,000 European-ancestry participants^30^. White blood cells are key components in the innate and adaptive immune responses to bacterial and viral infections^60^. Abdominal infections and gastroenteritis were associated with variants located in the *SLC35F* (lead SNP rs4333882, *P* = 2.14e−14) and *FSTL5* loci (rare variant, EAF cases = 0.0014, lead SNP rs115809651. *P* = 8.07e−10)*,* respectively. Although the biological functions of these genes are unknown, their associations with blood levels of cytokines (chemokines, interleukins, interferons)^29^ suggest potential importance for the innate immune response. Cytokines are key components in the biochemical pathways affecting migration and activation of white blood cells^60^ and are also fundamental in the biological processes of autoinflammatory diseases such as rheumatoid arthritis^61^ and inflammatory bowel diseases^62^.

Biologically plausible correlations were found between some of the infection phenotypes and chronic diseases, most frequently autoimmune diseases and cancer. While such co-morbidities increase the susceptibility for secondary infections, common genetic determinants that increase the risk for infections, inflammatory disease and malignancies could exist and be revealed either through studies of local genetic correlation or colocalization between traits. In this study, we observed colocalization of a variant associated with abdominal infections and gene expression in colon, suggesting causality of *PPP1R14A* in this class of infections.

This study has several strengths and limitations. To our knowledge, this is the largest interpreted GWAS to date on bacterial and viral infections using carefully determined compound phenotypes for important infection categories. External validation would have greatly added to the results but was not possible as other comparative data were unavailable. Replication using smaller biobanks with electronic health data would also be valuable to validate our findings. The definition of phenotypes based on specific diagnosis codes is a strength of this study, which is likely to increase sensitivity and specificity in relation to previous studies using self-reported history of diseases or ICD-10 codes without any curation. Still, some misclassifications are expected where the diagnosis set by the treating physician did not accurately describe the clinical syndrome; this situation may have resulted in false positive or negative cases and decreased the power of our analyses. It should be noted that there was sometimes an overlap in ICD-10 codes between phenotypes. As expected, there was some discrepancy in results between the combined phenotypes and subgroups (such as all RTIs vs. bacterial pneumonia). While the larger phenotypes are helpful to capture genetic variants related to the general systemic or local host immune defence, more specific phenotypes and larger cohorts may be required to find for example genetic determinants of pathogen-specific endothelial adhesion molecules. The conservative approach of refining the study cohort to correct for population structure and cryptic relatedness may have resulted in a lower estimated heritability. Further study is required to determine whether our observations result from genetic determinants affecting the risk for several disease groups or causal effects of co-morbidities that increase the vulnerability to infections.

## Conclusions

In conclusion, we report multiple novel loci associated with bacterial and viral infections in a large population cohort and provide interpretation of these results in the context of previous literature. Our results add significantly to the limited existing data and biological insights in this field. The genetic determinants of infectious disease susceptibility identified in this study could potentially be used to help identify target genes for the development of novel therapeutics for prevention or treatment of these diseases.

## Supplementary Information

Supplementary Figure S1.

Supplementary Figure S2.

Supplementary Figure S3.

Supplementary Tables.

## Data Availability

Data sets related to this article are available at UKB resource (https://www.ukbiobank.ac.uk/). GWAS summary statistics will be posted in GRASP (Genome-wide Repository of Associations between SNPs and Phenotypes; https://grasp.nhlbi.nih.gov).
