# Supplementary material for: A genome-wide association study in a large community-based cohort identifies multiple loci associated with susceptibility to bacterial and viral infections
Source: Sci Rep. 2022 Feb 16;12:2582. doi: 10.1038/s41598-022-05838-z (PMC8850418; doi:10.1038/s41598-022-05838-z)

Figure S1. Manhattan plots. Associations of genetic variants with infection phenotypes in the UK Biobank cohort (n=337,536) are shown. Negative log_{10}-transformed P-values for each SNP (y axis) are plotted by chromosomal position (x axis). The grey line represents the threshold for genome-wide statistically significant associations (P = 5e-08).

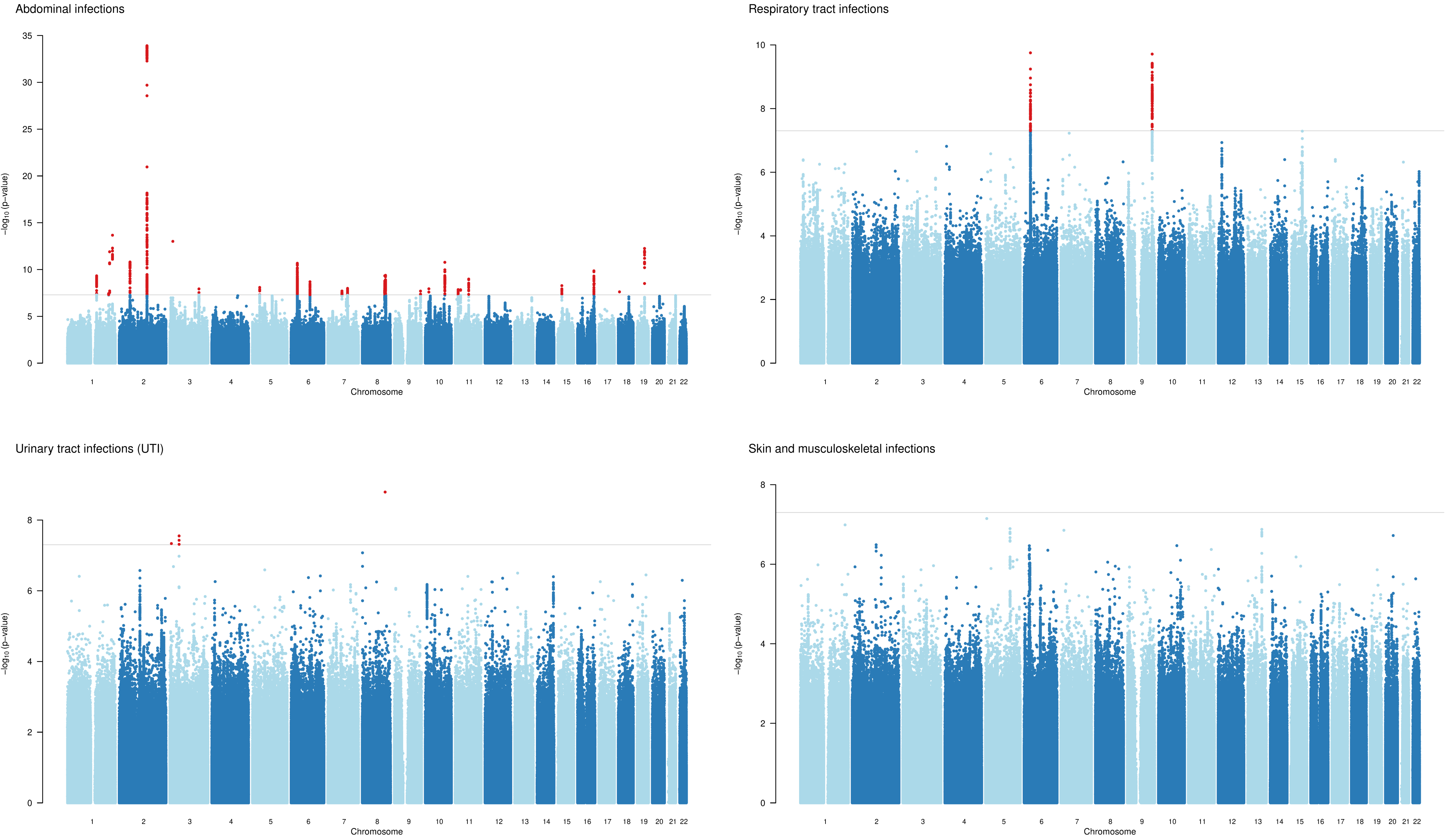
Abdominal infections
Respiratory tract infections
Urinary tract infections (UTI)
Skin and musculoskeletal infections
$-\log_{10}$ (p−value)
Chromosome
1 2 3 4 5 6 7 8 9 10 11 12 13 14 15 16 17 18 19 20 21 22

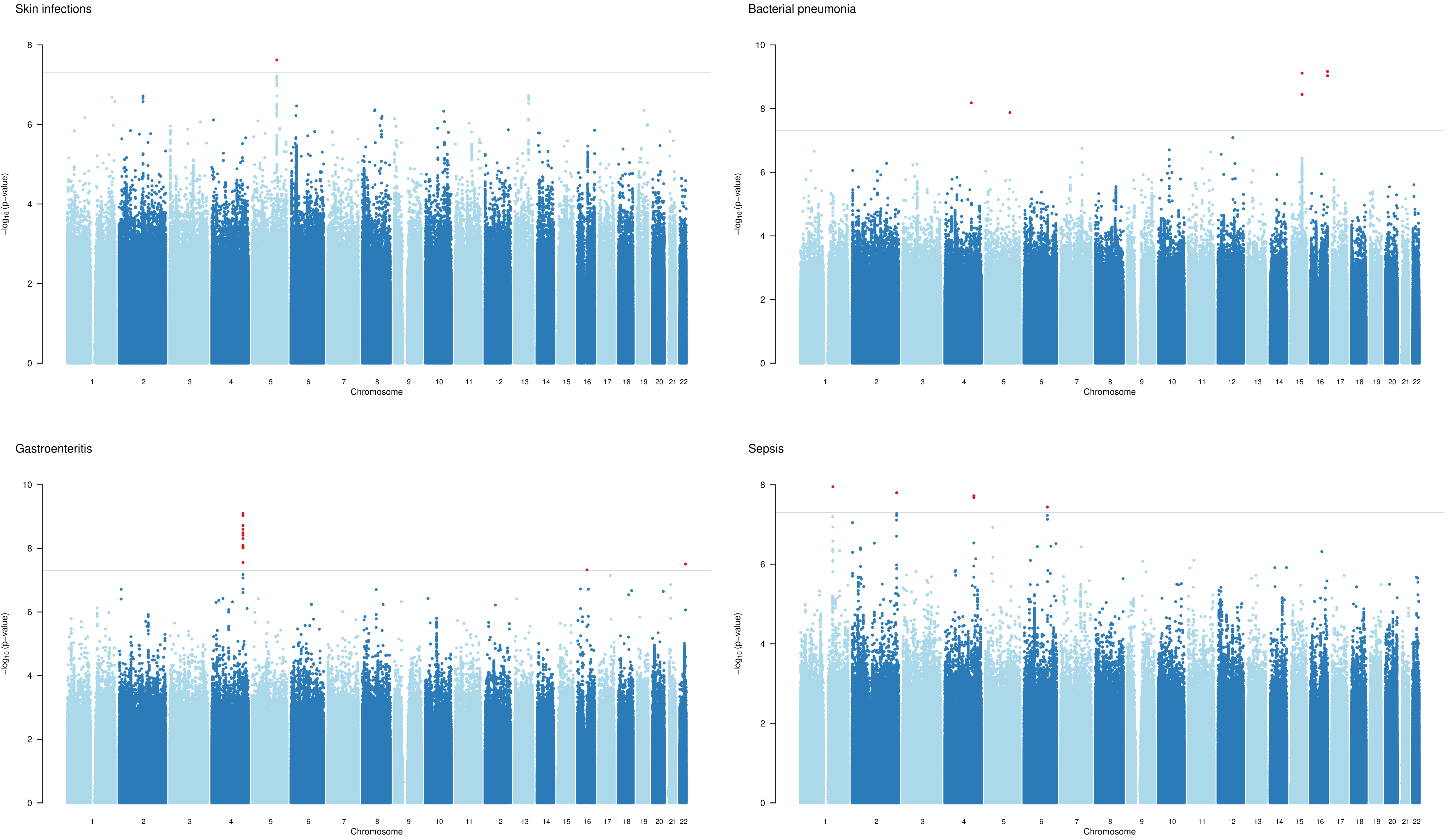
Skin infections
Bacterial pneumonia
Gastroenteritis
Sepsis
−log10 (p−value)
Chromosome
1
2
3
4
5
6
7
8
9
10
11
12
13
14
15
16
17
18
19
20
21
22

Specified viral infections

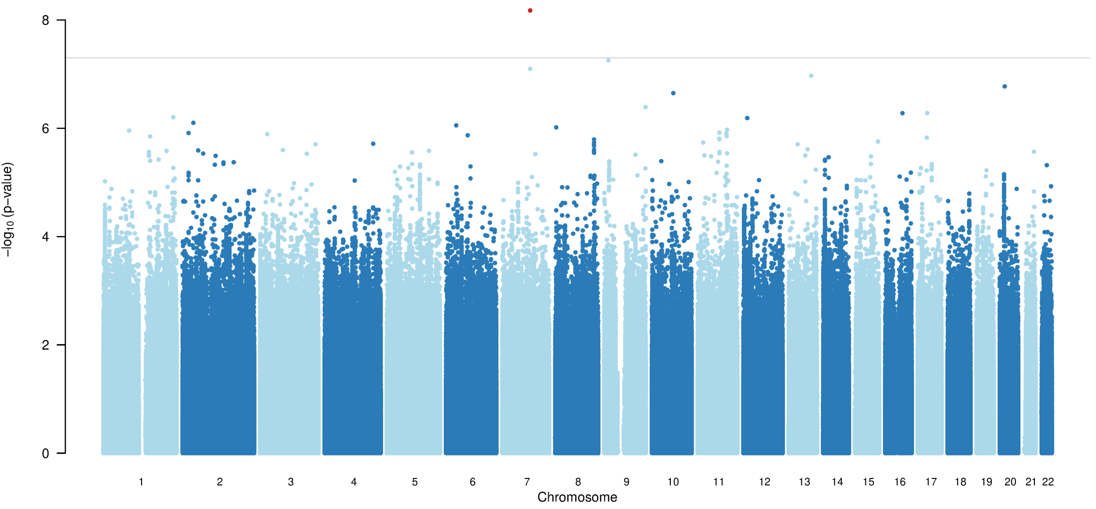

Bacterial gastroenteritis

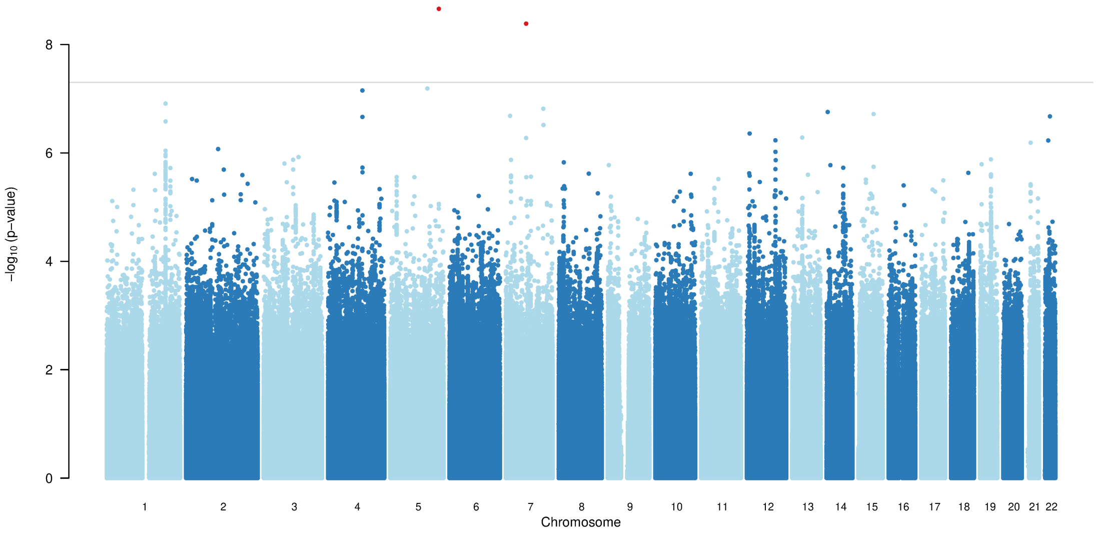

Urogenital (non-UTI) infections

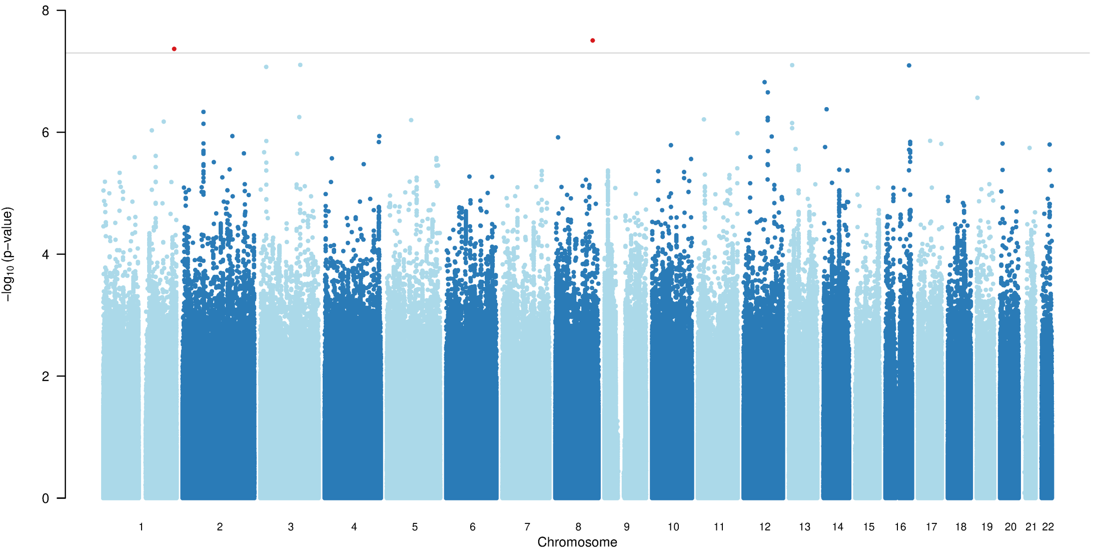

Cystitis

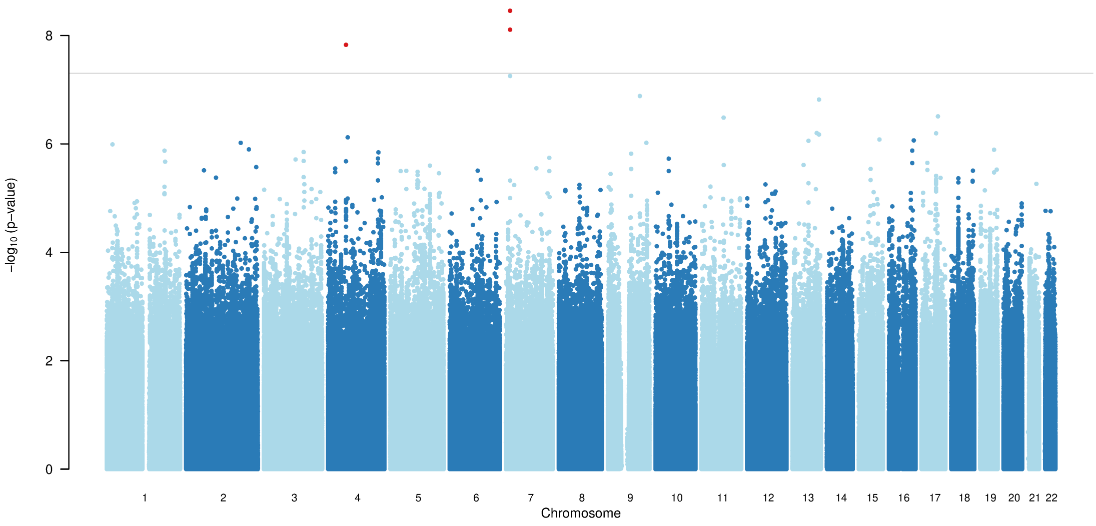

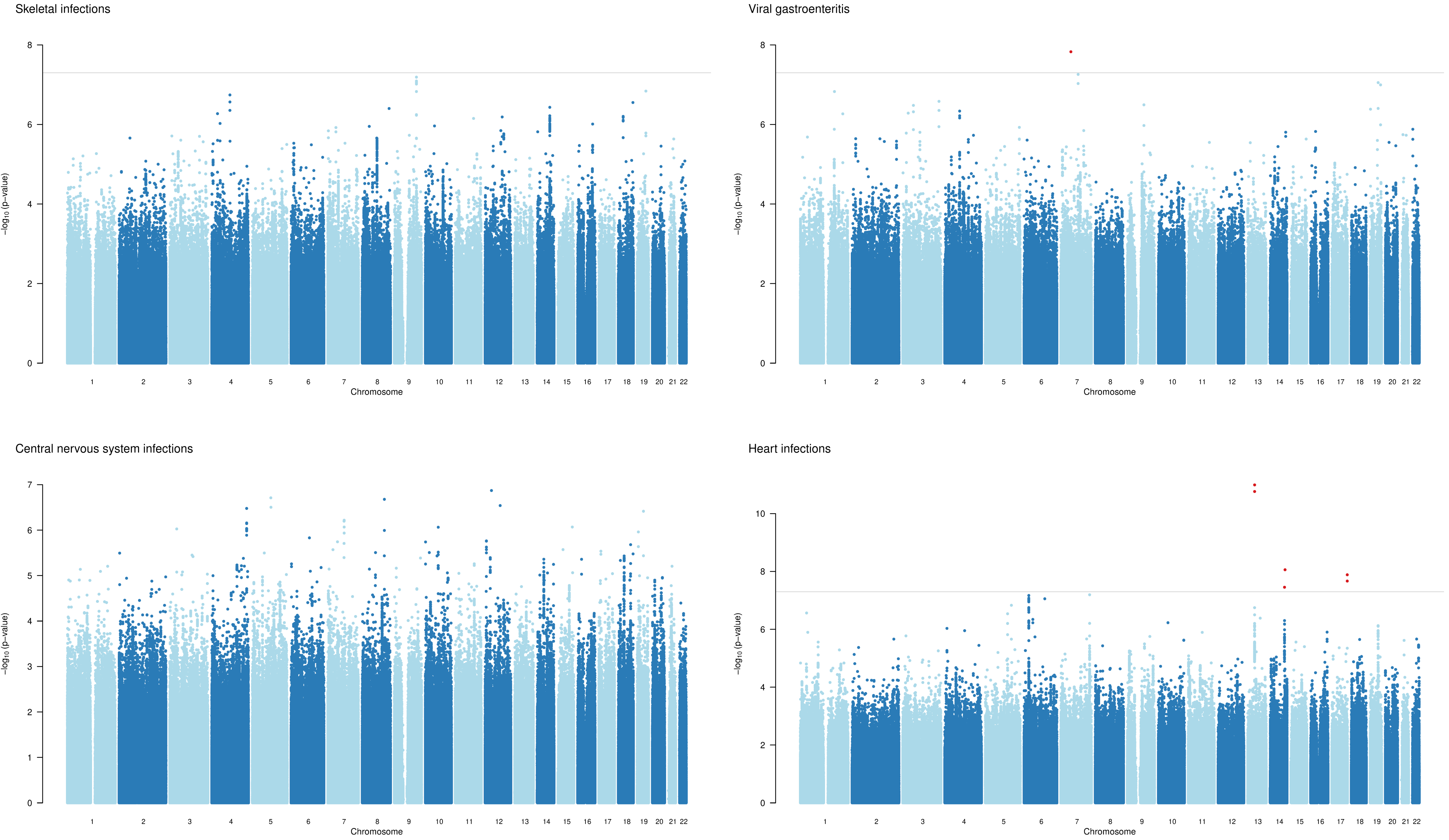

Skeletal infections
Viral gastroenteritis
Central nervous system infections
Heart infections
−log10 (p−value)
Chromosome
1 2 3 4 5 6 7 8 9 10 11 12 13 14 15 16 17 18 19 20 21 22

Influenza and viral pneumonia

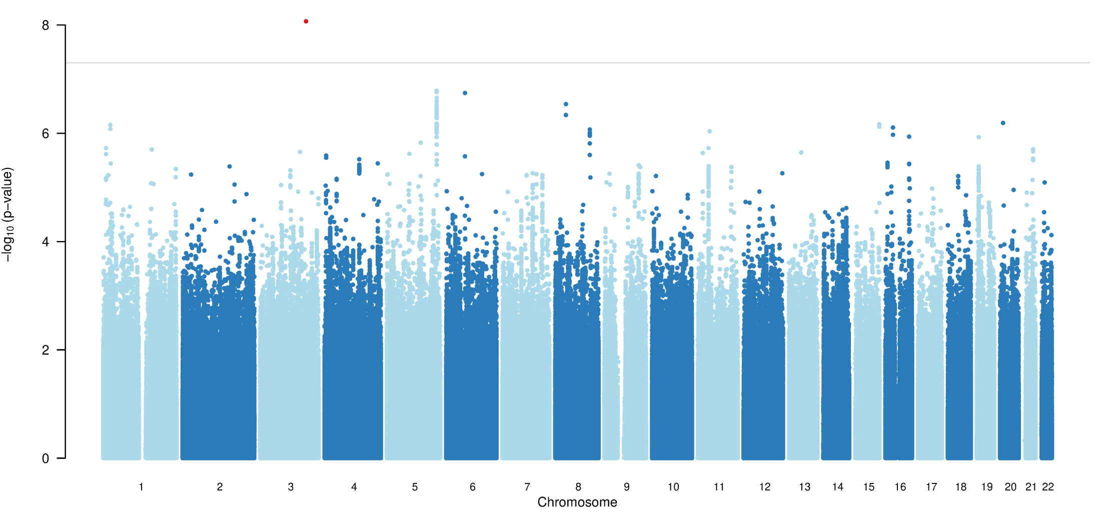

Sexually transmitted diseases

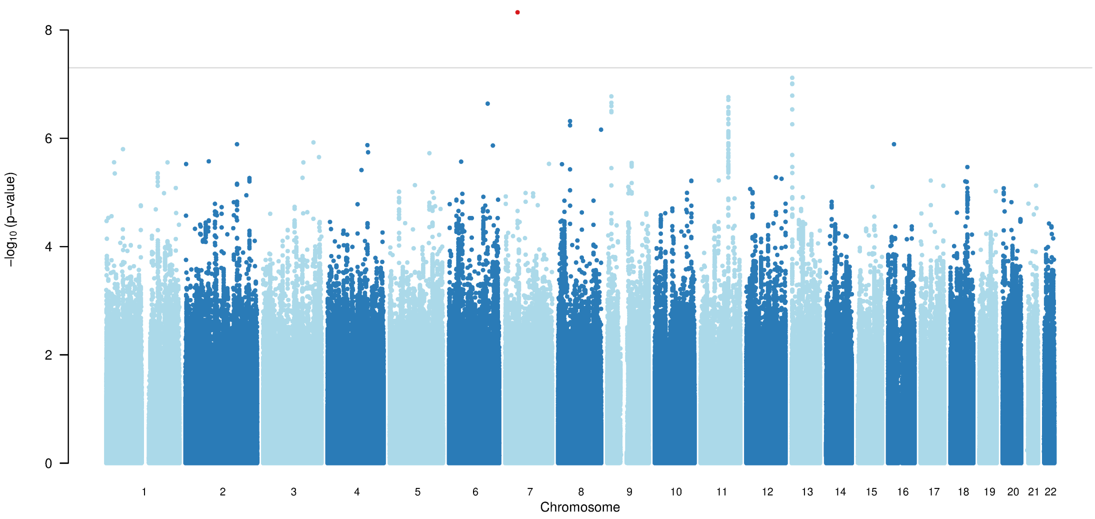

Supplement: Supplementary file 1 — Supplementary Figure S1. [file 41598_2022_5838_MOESM1_ESM.pdf]
