# Supplementary material for: A genome-wide association study in a large community-based cohort identifies multiple loci associated with susceptibility to bacterial and viral infections
Source: Sci Rep. 2022 Feb 16;12:2582. doi: 10.1038/s41598-022-05838-z (PMC8850418; doi:10.1038/s41598-022-05838-z)
Supplement: Supplementary file 2 — Supplementary Figure S2. [file 41598_2022_5838_MOESM2_ESM.pdf]

Figure S2. Quantile-quantile (QQ) plots. The expected under the null hypothesis *vs*. observed negative $\log_{10}$-transformed P-values for genome-wide association results for infection phenotypes in the UK Biobank cohort.

Abdominal infections

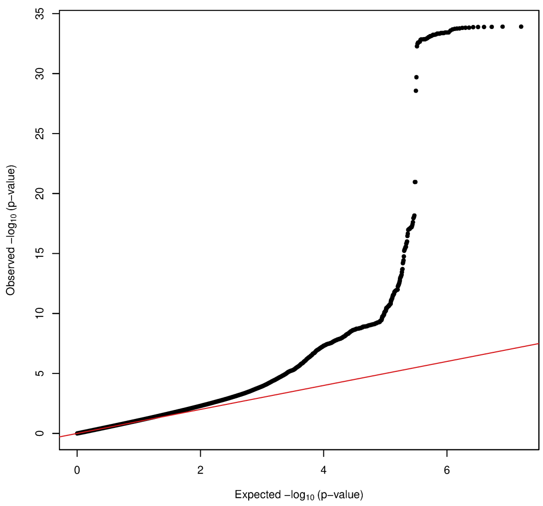

Respiratory tract infections

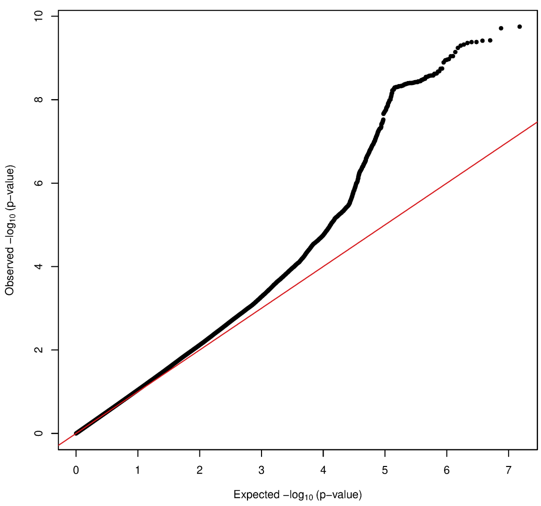

Urinary tract infections (UTI)

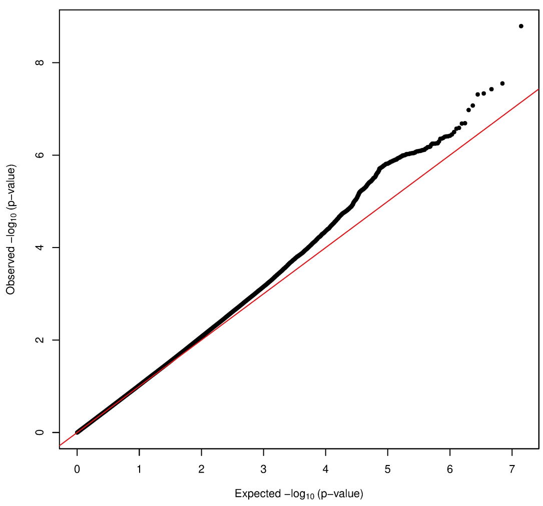

Skin and musculoskeletal infections

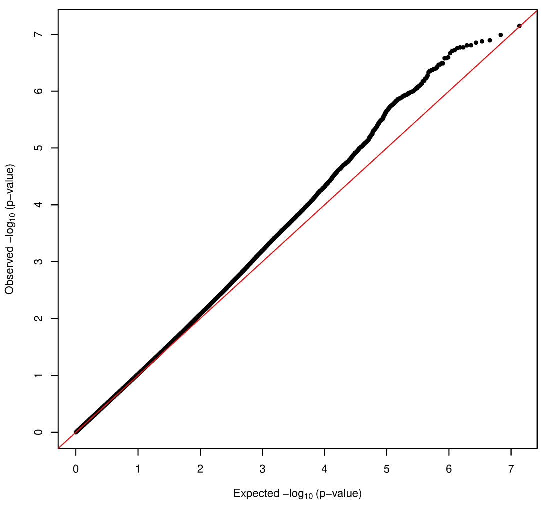

Skin infections

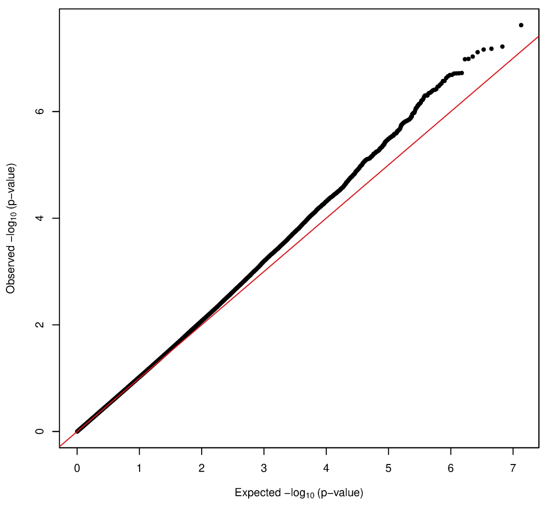

Bacterial pneumonia

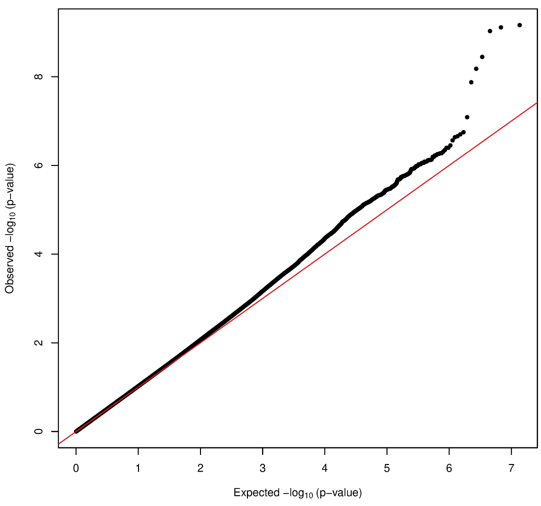

Gastroenteritis

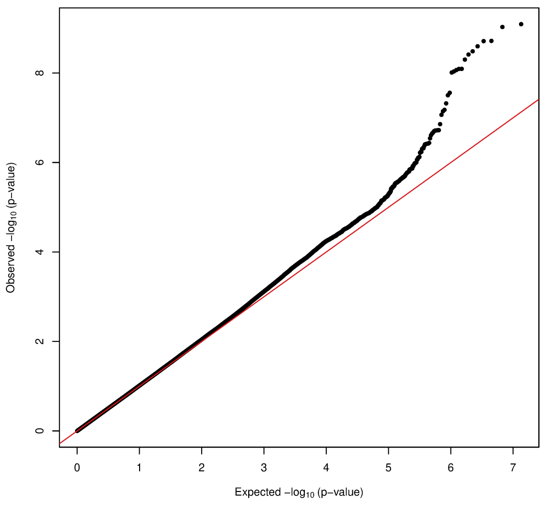

Sepsis

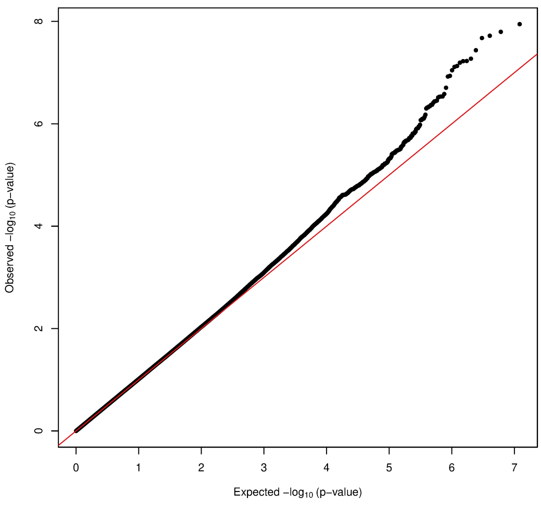

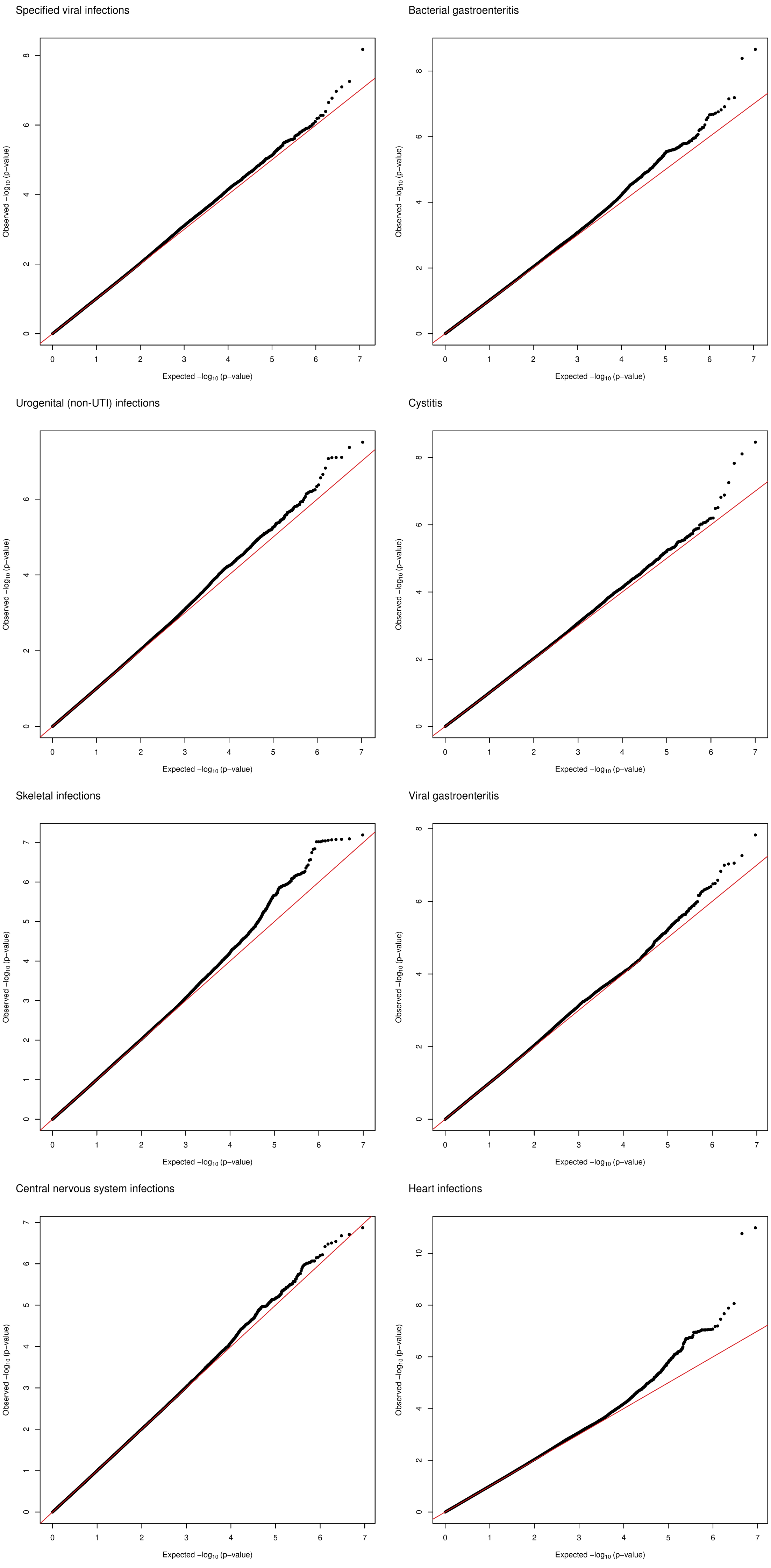

Specified viral infections
Bacterial gastroenteritis
Urogenital (non-UTI) infections
Cystitis
Skeletal infections
Viral gastroenteritis
Central nervous system infections
Heart infections
Observed −log10 (p−value)
Expected −log10 (p−value)

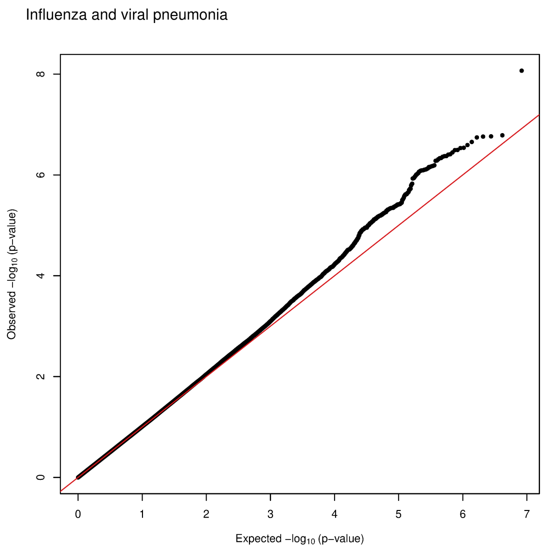
Influenza and viral pneumonia
Observed −log10 (p−value)
Expected −log10 (p−value)
0
2
4
6
8
0
1
2
3
4
5
6
7

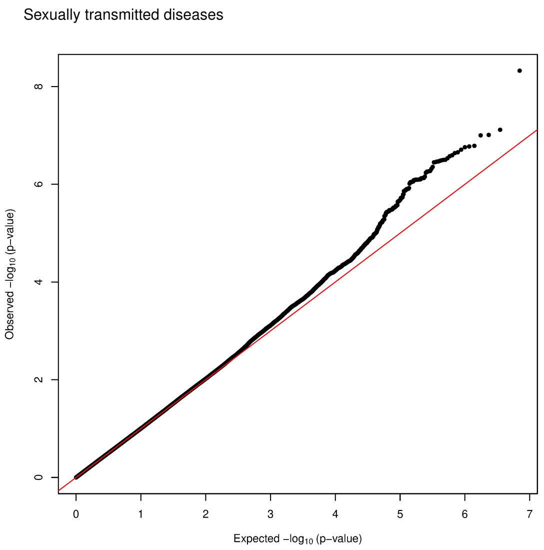
Sexually transmitted diseases
Observed −log10 (p−value)
Expected −log10 (p−value)
0
2
4
6
8
0
1
2
3
4
5
6
7
