# Supplementary material for: A genome-wide association study in a large community-based cohort identifies multiple loci associated with susceptibility to bacterial and viral infections
Source: Sci Rep. 2022 Feb 16;12:2582. doi: 10.1038/s41598-022-05838-z (PMC8850418; doi:10.1038/s41598-022-05838-z)
Supplement: Supplementary file 3 — Supplementary Figure S3. [file 41598_2022_5838_MOESM3_ESM.pdf]

Figure S3A. Phenome-wide associations between infection phenotypes of the present GWAS. “adj. n_{pheno} + n_{snps}“ refers to a Bonferroni-adjusted P-value based on the number of phenotypes and SNPs tested.

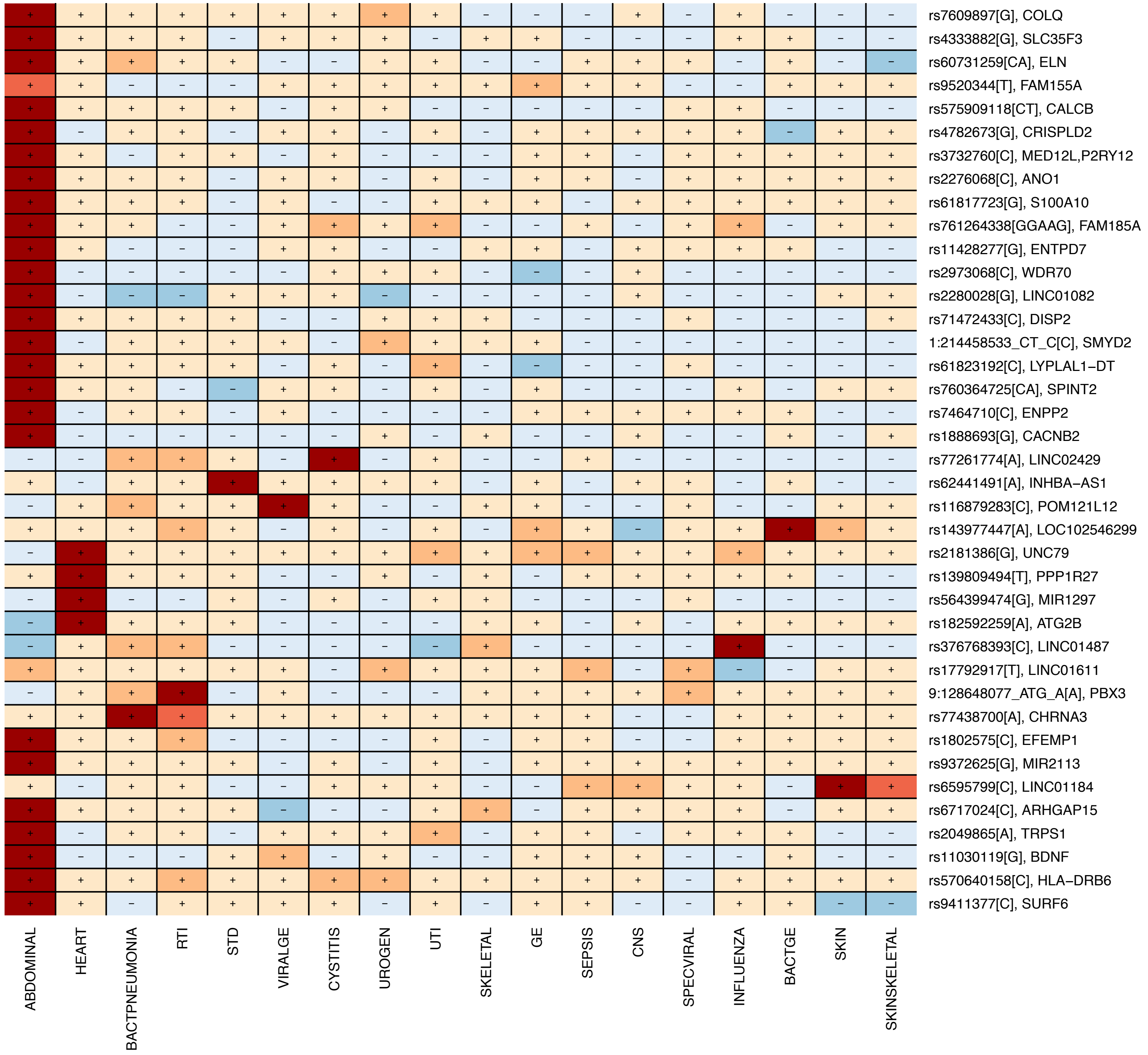

rs7609897[G], COLQ
rs4333882[G], SLC35F3
rs60731259[CA], ELN
rs9520344[T], FAM155A
rs575909118[CT], CALCB
rs4782673[G], CRISPLD2
rs3732760[C], MED12L,P2RY12
rs2276068[C], ANO1
rs61817723[G], S100A10
rs761264338[GGAAG], FAM185A
rs11428277[G], ENTPD7
rs2973068[C], WDR70
rs2280028[G], LINC01082
rs71472433[C], DISP2
1:214458533_CT_C[C], SMYD2
rs61823192[C], LYPLAL1−DT
rs760364725[CA], SPINT2
rs7464710[C], ENPP2
rs1888693[G], CACNB2
rs77261774[A], LINC02429
rs62441491[A], INHBA−AS1
rs116879283[C], POM121L12
rs143977447[A], LOC102546299
rs2181386[G], UNC79
rs139809494[T], PPP1R27
rs564399474[G], MIR1297
rs182592259[A], ATG2B
rs376768393[C], LINC01487
rs17792917[T], LINC01611
9:128648077_ATG_A[A], PBX3
rs77438700[A], CHRNA3
rs1802575[C], EFEMP1
rs9372625[G], MIR2113
rs6595799[C], LINC01184
rs6717024[C], ARHGAP15
rs2049865[A], TRPS1
rs11030119[G], BDNF
rs570640158[C], HLA−DRB6
rs9411377[C], SURF6
ABDOMINAL
HEART
BACTPNEUMONIA
RTI
STD
VIRALGE
CYSTITIS
UROGEN
UTI
SKELETAL
GE
SEPSIS
CNS
SPECVIRAL
INFLUENZA
BACTGE
SKIN
SKINSKELETAL

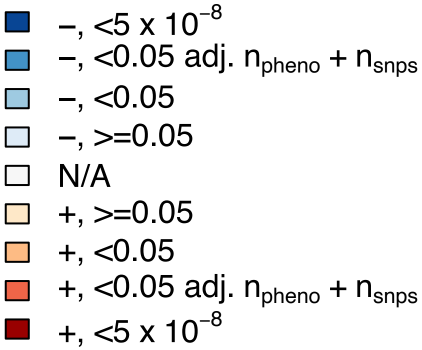
−, <5 x 10^{-8}
−, <0.05 adj. $n_{pheno} + n_{snps}$
−, <0.05
−, >=0.05
N/A
+, >=0.05
+, <0.05
+, <0.05 adj. $n_{pheno} + n_{snps}$
+, <5 x 10^{-8}

Figure S3B. Phenome-wide associations between shared significant loci across infectious and non-infectious phenotypes in the UK Biobank cohort.

“adj. n_{pheno} + n_{snps}“ refers to a Bonferroni-adjusted P-value based on the number of phenotypes and SNPs tested.

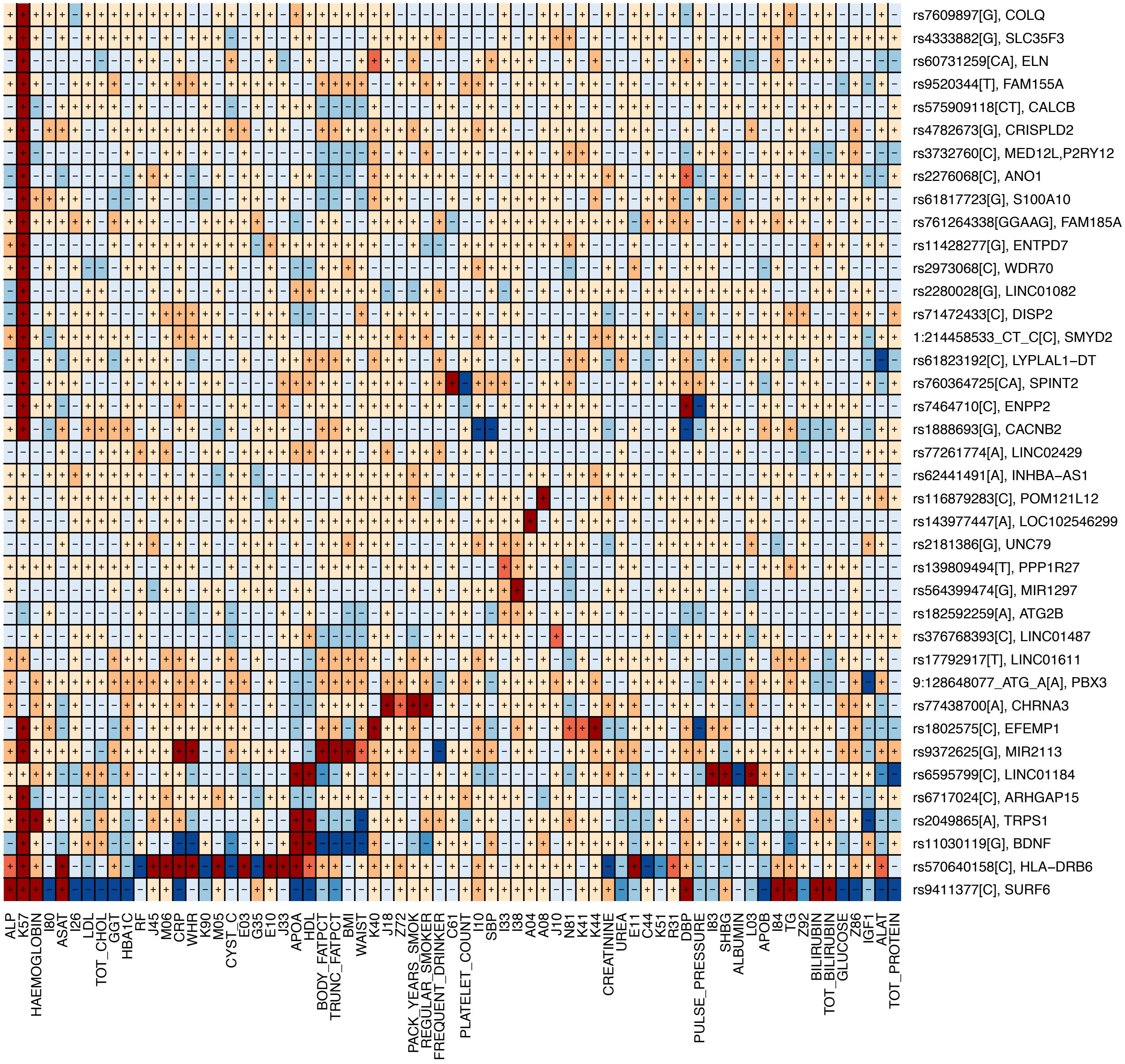

rs7609897[G], COLQ
rs4333882[G], SLC35F3
rs60731259[CA], ELN
rs9520344[T], FAM155A
rs575909118[CT], CALCB
rs4782673[G], CRISPLD2
rs3732760[C], MED12L,P2RY12
rs2276068[C], ANO1
rs61817723[G], S100A10
rs761264338[GGAAG], FAM185A
rs11428277[G], ENTPD7
rs2973068[C], WDR70
rs2280028[G], LINC01082
rs71472433[C], DISP2
1:214458533_CT_C[C], SMYD2
rs61823192[C], LYPLAL1−DT
rs760364725[CA], SPINT2
rs7464710[C], ENPP2
rs1888693[G], CACNB2
rs77261774[A], LINC02429
rs62441491[A], INHBA−AS1
rs116879283[C], POM121L12
rs143977447[A], LOC102546299
rs2181386[G], UNC79
rs139809494[T], PPP1R27
rs564399474[G], MIR1297
rs182592259[A], ATG2B
rs376768393[C], LINC01487
rs17792917[T], LINC01611
9:128648077_ATG_A[A], PBX3
rs77438700[A], CHRNA3
rs1802575[C], EFEMP1
rs9372625[G], MIR2113
rs6595799[C], LINC01184
rs6717024[C], ARHGAP15
rs2049865[A], TRPS1
rs11030119[G], BDNF
rs570640158[C], HLA−DRB6
rs9411377[C], SURF6
ALP
K57
HAEMOGLOBIN
I80
ASAT
I26
LDL
TOT_CHOL
GGT
HBA1C
RF
J45
M06
CRP
WHR
K90
M05
CYST_C
E03
G35
E10
J33
APOA
HDL
BODY_FATPCT
TRUNC_FATPCT
BMI
WAIST
K40
J18
Z72
PACK_YEARS_SMOK
REGULAR_SMOKER
FREQUENT_DRINKER
C61
PLATELET_COUNT
I10
SBP
I33
I38
A04
A08
J10
N81
K41
K44
CREATININE
UREA
E11
C44
K51
R31
DBP
PULSE_PRESSURE
I83
SHBG
ALBUMIN
L03
APOB
I84
TG
Z92
BILIRUBIN
TOT_BILIRUBIN
GLUCOSE
Z86
IGF1
ALAT
TOT_PROTEIN

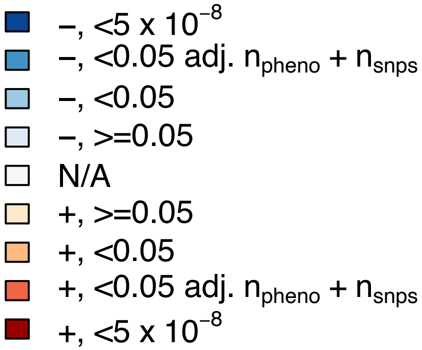

−, <5 x 10^−8
−, <0.05 adj. n_pheno + n_snps
−, <0.05
−, >=0.05
N/A
+, >=0.05
+, <0.05
+, <0.05 adj. n_pheno + n_snps
+, <5 x 10^−8
