# Supplementary material for: A genome-wide association study in a large community-based cohort identifies multiple loci associated with susceptibility to bacterial and viral infections
Source: Sci Rep. 2022 Feb 16;12:2582. doi: 10.1038/s41598-022-05838-z (PMC8850418; doi:10.1038/s41598-022-05838-z)
Supplement: Supplementary file 4 — Supplementary Tables. [file 41598_2022_5838_MOESM4_ESM.docx]

**Table S1. International Classification of Disease (ICD)-10 diagnose codes per infection phenotypes and subgroups.** (Version:2015, available at http://www.who.int)

| **Phenotype** | **ICD-10 diagnose codes** |
| --- | --- |
| Abdominal infections | *//D73.3, K35-37, K57, K61, K63.0, K65, K75.0, K81, K83.0*  D73.3 Abscess of spleen K35 Acute appendicitis K36 Other appendicitis K37 Unspecified appendicitis K57 Diverticular disease of intestine K61 Abscess of anal and rectal regions K63.0 Abscess of intestine K65 Peritonitis K75.0 Abscess of liver K81 Cholecystitis K83.0 Cholangitis |
| Central nervous system infections | *//A17, A20.3, A32.1, A39.0, A80-89, B00.3, B00.4, B01.0, B01.1, B02.0, B02.1, B02.2, B05.0, B05.1, B06.0, B26.1, B26.2, G00, G01, G02.0, G04-07*  A17 Tuberculosis of nervous system A20.3 Plague meningitis A32.1 Listerial meningitis and meningoencephalitis A39.0 Meningococcal meningitis A80 Acute poliomyelitis A81 Atypical virus infections of central nervous system A82 Rabies A83 Mosquito-borne viral encephalitis A84 Tick-borne viral encephalitis A85 Other viral encephalitis, not elsewhere classified A86 Unspecified viral encephalitis A87 Viral meningitis A88 Other viral infections of central nervous system, not elsewhere classified A89 Unspecified viral infection of central nervous system B00.3 Herpesviral meningitis B00.4 Herpesviral encephalitis B01.0 Varicella meningitis B01.1 Varicella encephalitis B02.0 Zoster encephalitis B02.1 Zoster meningitis B02.2 Zoster with other nervous system involvement B05.0 Measles complicated by encephalitis B05.1 Measles complicated by meningitis B06.0 Rubella with neurological complications B26.1 parotitis with meningitis B26.2 Mumps encephalitis G00 Bacterial meningitis, not elsewhere classified G01 Meningitis in bacterial diseases classified elsewhere G02.0 Meningitis in viral diseases classified elsewhere G04 Encephalitis, myelitis and encephalomyelitis G05 Encephalitis, myelitis and encephalomyelitis in diseases classified elsewhere G06 Intracranial and intraspinal abscess and granuloma G07 Intracranial and intraspinal abscess and granuloma in diseases classified elsewhere |
| Gastroenteritis | *a. Bacterial gastroenteritis //A00-A04*  A00 Cholera A01 Typhoid and paratyphoid fevers A02 Other salmonella infections A03 Shigellosis A04 Other bacterial intestinal infections  *b. Viral gastroenteritis //A08*  A08 Viral and other specified intestinal infections  *Other //A09*  A09 Other gastroenteritis and colitis of infectious and unspecified origin |
| Heart infections | *//A39.5, I30.1, I32.0, I33, I38, B33.2, I40.0, I41.0, I41.1*  A39.5 Meningococcal heart disease I30.1 Infective pericarditis I32.0 Pericarditis in bacterial diseases classified elsewhere I33 Acute and subacute endocarditis I38 Endocarditis, valve unspecified I40.0 Infective myocarditis I41.0 Myocarditis in bacterial diseases classified elsewhere I41.1 Myocarditis in viral diseases classified elsewhere |
| Respiratory tract infections | *a. Bacterial pneumonia //A48.1, A70, J13, J14, J15, J16, J18*  A48.1 Legionnaires disease A70 Chlamydia psittaci infection J13 Pneumonia due to Streptococcus pneumoniae J14 Pneumonia due to Haemophilus influenzae J15 Bacterial pneumonia, not elsewhere classified J16 Pneumonia due to other infectious organisms, not elsewhere classified J17 Pneumonia in diseases classified elsewhere J18 Pneumonia, organism unspecified  *b. Influenza and viral pneumonia //J09-J12*  J09 Influenza due to certain identified influenza virus J10 Influenza due to other identified influenza virus J11 Influenza, virus not identified J12 Viral pneumonia, not elsewhere classified  *Other //A15, A16, A20.2, A21.2, A22.1, A31.0, A36.0, A36.1, A36.2, A37, A38, B01.2, B05.2, B05.3, B25.0, J01-06, J17, J20-22, J32, J36.9, J39.0, J39.1, J40, J44.0, J44.1, J85.1, J85.2, J85.3, J86, H66*  A15 Respiratory tuberculosis, bacteriologically and histologically confirmed A16 Respiratory tuberculosis, not confirmed bacteriologically or histologically A20.2 Pneumonic plague A21.2 Pulmonary tularaemia A22.1 Pulmonary anthrax A31.0 Pulmonary mycobacterial infection A36.0 Pharyngeal diphtheria A36.1 Nasopharyngeal diphtheria A36.2 Laryngeal diphtheria A37 Whooping cough A38 Scarlet fever B01.2 Varicella pneumonia B05.2 Measles complicated by pneumonia B05.3 Measles complicated by otitis media B25.0 Cytomegaloviral pneumonitis J01 Acute sinusitis J02 Acute pharyngitis J03 Acute tonsillitis J04 Acute laryngitis and tracheitis J05 Acute obstructive laryngitis [croup] and epiglottitis J06 Acute upper respiratory infections of multiple and unspecified sites J17 Pneumonia in diseases classified elsewhere J20 Acute bronchitis J21 Acute bronchiolitis J22 Unspecified acute lower respiratory infection J32 Chronic sinusitis J36.9 Peritonsillar abscess J39.0 Retropharyngeal and parapharyngeal abscess J39.1 Other abscess of pharynx J40 Bronchitis, not specified as acute or chronic J44.0 Chronic obstructive pulmonary disease with acute lower respiratory infection J44.1 Chronic obstructive pulmonary disease with acute exacerbation, unspecified J85.1 Abscess of lung with pneumonia J85.2 Abscess of lung without pneumonia J85.3 Abscess of mediastinum J86 Pyothorax H66 Suppurative and unspecified otitis media |
| Sepsis | *//A39.2, A40, A41*  A39.2 Acute meningococcaemia A40 Streptococcal sepsis A41 Other sepsis |
| Sexually transmitted diseases | *//A50-58, A60, A63-64*  A50 Congenital syphilis A51 Early syphilis A52 Late syphilis A53 Other and unspecified syphilis A54 Gonococcal infection A55 Chlamydial lymphogranuloma (venereum) A56 Other sexually transmitted chlamydial diseases A57 Chancroid A58 Granuloma inguinale A60 Anogenital herpesviral [herpes simplex] infection A63 Other predominantly sexually transmitted diseases, not elsewhere classified A64 Unspecified sexually transmitted disease |
| Skin and musculoskeletal infections | *a. Skin infections //A36.3, A46, J34.0, L00-L05, L08*  A36.3 Cutaneous diphtheria A46 Erysipelas J34.0 Abscess, furuncle and carbuncle of nose L00 Staphylococcal scalded skin syndrome L01 Impetigo L02 Cutaneous abscess, furuncle and carbuncle L03 Cellulitis L04 Acute lymphadenitis L05 Pilonidal cyst L08 Other local infections of skin and subcutaneous tissue  *b. Musculoskeletal infections //B33.0, M00, M01.0, M01.1, M01.3, M01.4, M01.5, M46.2, M46.3, M46.5, M49.0, M49.1, M49.2, M60.0, M65.0, M65.1, M71.0, M71.1, M86*  B33.0 Epidemic myalgia M00 Pyogenic arthritis M01.0 Meningococcal arthritis M01.1 Tuberculous arthritis M01.3 Arthritis in other bacterial diseases classified elsewhere M01.4 Rubella arthritis M01.5 Arthritis in other viral diseases classified elsewhere M46.2 Osteomyelitis of vertebra M46.3 Infection of intervertebral disc (pyogenic) M46.5 Other infective spondylopathies M49.0 Tuberculosis of spine M49.1 Brucella spondylitis M49.2 Enterobacterial spondylitis M60.0 Infective myositis M65.0 Abscess of tendon sheath M65.1 Other infective (teno)synovitis M71.0 Abscess of bursa M71.1 Other infective bursitis M86 Osteomyelitis |
| Specified viral infections | *//A90-99, B00-06, B08, B09, B15-19, B20-27, B30, B33, B34, B97*  A90 Dengue fever [classical dengue] A91 Dengue haemorrhagic fever A92 Other mosquito-borne viral fevers A93 Other arthropod-borne viral fevers, not elsewhere classified A94 Unspecified arthropod-borne viral fever A95 Yellow fever A96 Arenaviral haemorrhagic fever A98 Other viral haemorrhagic fevers, not elsewhere classified A99 Unspecified viral haemorrhagic fever B00 Herpesviral [herpes simplex] infections B01 Varicella [chickenpox] B02 Zoster [herpes zoster] B03 Smallpox B04 Monkeypox B05 Measles B06 Rubella [German measles] B08 Other viral infections characterized by skin and mucous membrane lesions, not elsewhere classified B09 Unspecified viral infection characterized by skin and mucous membrane lesions B15 Acute hepatitis A B16 Acute hepatitis B B17 Other acute viral hepatitis B18 Chronic viral hepatitis B19 Unspecified viral hepatitis B20 Human immunodeficiency virus [HIV] disease resulting in infectious and parasitic diseases B21 Human immunodeficiency virus [HIV] disease resulting in malignant neoplasms B22 Human immunodeficiency virus [HIV] disease resulting in other specified diseases B23 Human immunodeficiency virus [HIV] disease resulting in other conditions B24 Unspecified human immunodeficiency virus [HIV] disease B25 Cytomegaloviral disease B26 Mumps B27 Infectious mononucleosis B30 Viral conjunctivitis B33 Other viral diseases, not elsewhere classified B34 Viral infection of unspecified site B97 Viral agents as the cause of diseases classified to other chapters |
| Urinary tract infections (UTI) | *a. Cystitis //N30.0, N30.9*  N30.0 Acute cystitis N30.9 Cystitis, unspecified  *b. Pyelonephritis //N10*  N10 Acute tubulo-interstitial nephritis  *Other //N15.1, N39.0, N41.0, N41.3*  N15.1 Renal and perinephric abscess N39.0 Urinary tract infection, site not specified N41.0 Acute prostatitis N41.3 Prostatocystitis |
| Urogenital (non-UTI) infections | *//N34.0, N41.1, N41.2, N43.1, N45, N76.0, N76.2, N76.4*  N34.0 Urethral abscess N41.1 Chronic prostatitis N41.2 Abscess of prostate N43.1 Infected hydrocele N45 Orchitis and epididymitis N76.0 Acute vaginitis N76.2 Acute vulvitis N76.4 Abscess of vulva |

**Table S2. Tissues included in the extraction of data from expression quantitative trait locus (eQTL) studies used for functional annotation.**

| **Phenotype** | **Tissues** |
| --- | --- |
| All phenotypes | Spleen, whole blood. |
| Abdominal infections | Visceral adipose, sigmoid colon, transverse colon, liver, pancreas, small intestine - terminal ileum. |
| Gastroenteritis | Sigmoid colon, transverse colon, esophagus - gastroesophageal junction, esophagus – mucosa, esophagus - muscularis, stomach. |
| Heart infections | Heart - atrial appendage, heart - left ventricle. |
| Respiratory tract infections | Lung. |
| Sexually transmitted diseases | Ectocervix, endocervix, fallopian tube, prostate. testis, uterus, vagina. |
| Skin and musculoskeletal infections | Subcutaneous adipose, EBV-transformed fibroblast cells, skeletal muscle, suprapubic skin-not sun exposed, lower leg skin – sun exposed. |
| Urinary tract infections (UTI) | Bladder, kidney - cortex, prostate. |
| Urogenital (non-UTI) infections | Ectocervix, endocervix, fallopian tube, prostate, testis, uterus, vagina. |

**Table S3. Genomic control lambda values of the GWAS analysis.**

| **Phenotype** | **lambda (all)** | **lambda (MAF > 1)** |
| --- | --- | --- |
| Abdominal infections | 1.101 | 1.166 |
| Central nervous system infections | 0.993 | 0.988 |
| Gastroenteritis | 1.020 | 1.022 |
| Bacterial gastroenteritis | 1.015 | 1.016 |
| Viral gastroenteritis | 1.008 | 0.990 |
| Heart infections | 1.004 | 1.002 |
| Respiratory tract infections | 1.056 | 1.080 |
| Bacterial pneumonia | 1.033 | 1.041 |
| Influenza and viral pneumonia | 1.007 | 1.007 |
| Sepsis | 1.024 | 1.031 |
| Sexually transmitted diseases | 1.009 | 1.009 |
| Skin and musculoskeletal infections | 1.028 | 1.036 |
| Skeletal infections | 0.996 | 0.985 |
| Skin infections | 1.030 | 1.040 |
| Specified viral infections | 1.010 | 1.010 |
| Urinary tract infections (UTI) | 1.028 | 1.037 |
| Cystitis | 1.003 | 1.000 |
| Urogenital (non-UTI) infections | 1.012 | 1.012 |

**Table S4. Results of fine mapping of the human leucocyte antigen (HLA) region.** Dosages for all possible alleles at each HLA locus were tested in separate logistic regression models. Only associations reaching the significance threshold of P < 1.6e-5 are presented.

| **Phenotype** | **Locus allele** | **EAF controls** | **EAF cases** | **Odds ratio (95% CI)** | **Beta** | **SE** | **P** |
| --- | --- | --- | --- | --- | --- | --- | --- |
| Abdominal infections | HLA-DRB4*01:03 | 0.256 | 0.265 | 1.055 (1.036-1.074) | 0.053 | 0.009 | 9.04e-09 |
| Abdominal infections | HLA-DRB4*99:01 | 0.650 | 0.639 | 0.954 (0.938-0.970) | -0.047 | 0.008 | 1.93e-08 |
| Abdominal infections | HLA-DQA1*03:01 | 0.203 | 0.210 | 1.054 (1.033-1.074) | 0.052 | 0.010 | 1.49e-07 |
| Abdominal infections | HLA-DRB1*04:01 | 0.114 | 0.119 | 1.064 (1.038-1.091) | 0.062 | 0.013 | 9.17e-07 |
| Respiratory tract infections | HLA-DQA1*03:01 | 0.203 | 0.213 | 1.072 (1.047-1.097) | 0.069 | 0.012 | 4.45e-09 |
| Respiratory tract infections | HLA-DRB1*04:01 | 0.114 | 0.120 | 1.073 (1.042-1.105) | 0.071 | 0.015 | 2.57e-06 |
| Respiratory tract infections | HLA-DQA1*02:01 | 0.145 | 0.138 | 0.938 (0.913-0.964) | -0.064 | 0.014 | 4.63e-06 |
| Respiratory tract infections | HLA-DRB1*07:01 | 0.146 | 0.138 | 0.939 (0.914-0.965) | -0.063 | 0.014 | 6.88e-06 |

**Table S5. Results reaching FDR < 0.01 in the colocalization analyses of SNP associations and gene expression (eQTL) data.** FDR is the false discovery rate (Benjamini-Yekutieli) calculated per phenotype and beta is the effect size in the Summary data-based Mendelian Randomization (SMR) test. P-values are presented for the SMR and HEIDI (HEterogeneity In Dependent Instruments) tests.

| **Phenotype** | **Tissue** | **Gene** | **beta (SMR)** | **se (SMR)** | **P-value (SMR)** | **FDR** | **P-value (HEIDI)** | **No. of SNPs (HEIDI)** |
| --- | --- | --- | --- | --- | --- | --- | --- | --- |
| Abdominal infections | Colon, sigmoid | PPP1R14A | 0.0878 | 0.0141 | 4.680e-10 | 1.79e-05 | 0.6010 | 20 |
|  | Adipose visceral omentum | HLA-DRB6 | 0.0398 | 0.0077 | 2.622-07 | 0.0012 | 0.1602 | 20 |
|  | Adipose visceral omentum | HLA-DQA2 | 0.0338 | 0.0065 | 2.022e-07 | 0.0012 | 0.2540 | 20 |
|  | Colon, sigmoid | HLA-DRB6 | 0.0404 | 0.0078 | 2.675e-07 | 0.0012 | 0.5566 | 20 |
|  | Colon, transverse | HLA-DQA2 | 0.0475 | 0.0089 | 1.231e-07 | 0.0012 | 0.01491 | 20 |
|  | Colon, transverse | PPP1R14A | 0.2578 | 0.0493 | 1.740e-07 | 0.0012 | 0.6974 | 17 |
|  | Whole blood | HLA-DRB6 | 0.0581 | 0.0112 | 2.431e-07 | 0.0012 | 0.0067 | 20 |
|  | Whole blood | HLA-DQA2 | 0.0408 | 0.0078 | 1.758e-07 | 0.0012 | 0.2258 | 20 |
|  | Adipose visceral omentum | DISP2 | 0.0503 | 0.0099 | 4.539e-07 | 0.0015 | 0.3920 | 20 |
|  | Colon, sigmoid | HLA-DQA2 | 0.0335 | 0.0066 | 4.480e-07 | 0.0015 | 0.4197 | 20 |
|  | Colon, transverse | HLA-DRB6 | 0.0493 | 0.0097 | 3.773e-07 | 0.0015 | 0.0252 | 20 |
|  | Spleen | HLA-DRB6 | 0.0392 | 0.0078 | 5.681e-07 | 0.0015 | 0.8905 | 20 |
|  | Spleen | HLA-DQA2 | 0.0384 | 0.0076 | 5.115e-07 | 0.0015 | 0.3566 | 20 |
|  | Whole blood | HLA-DRB1 | -0.2262 | 0.0451 | 5.497e-07 | 0.0015 | 0.0460 | 20 |
|  | Pancreas | DISP2 | 0.0496 | 0.0101 | 9.317e-07 | 0.0022 | 0.3774 | 20 |
|  | Adipose visceral omentum | ABO | -0.0598 | 0.0123 | 1.195e-06 | 0.0023 | 0.8227 | 20 |
|  | Colon, sigmoid | DISP2 | 0.0586 | 0.0121 | 1.272e-06 | 0.0023 | 0.2804 | 20 |
|  | Small intestine-terminal ileum | HLA-DQA2 | 0.0377 | 0.0078 | 1.266e-06 | 0.0023 | 0.3738 | 20 |
|  | Liver | HLA-DRB6 | 0.0432 | 0.0090 | 1.650e-06 | 0.0028 | 0.2888 | 20 |
|  | Colon, transverse | NOV | -0.1069 | 0.0223 | 1.751e-06 | 0.0029 | 0.4255 | 20 |
|  | Whole blood | PPIP5K2 | 0.1586 | 0.0332 | 1.857e-06 | 0.0029 | 0.1312 | 20 |

Abbreviations: se, standard error.

**Table S6. Estimated heritability for infection phenotypes with at least 5,000 cases.** h^2^ is the narrow-sense heritability estimate explained by the GWAS summary statistics from imputed variants (LDSC) or the additive genetics of genotyped variants (GCTA). The GCTA estimates are from a Haseman-Elston regression with standard errors computed using the Jackknife approach.

| **Phenotype** | **LDSC** | | **GCTA** | | |
| --- | --- | --- | --- | --- | --- |
|  | **h^2^** | **se** | **h^2^** | **se** | **P-value** |
| Abdominal infections | 0.0332 | 0.0024 | 0.0444 | 0.0024 | 5.32e-72 |
| Gastroenteritis | 0.0009 | 0.0014 | 0.0063 | 0.0018 | 5.59e-4 |
| Respiratory tract infections | 0.0106 | 0.0016 | 0.0224 | 0.0022 | 2.90e-23 |
| Bacterial pneumonia | 0.0054 | 0.0015 | 0.0082 | 0.0019 | 1.53e-05 |
| Skin and musculoskeletal infections | 0.0061 | 0.0017 | 0.0110 | 0.0021 | 2.52e-07 |
| Skin infections | 0.0066 | 0.0018 | 0.0107 | 0.0020 | 3.43e-07 |
| Urinary tract infections (UTI) | 0.0033 | 0.0016 | 0.0115 | 0.0022 | 3.34e-07 |

Abbreviations: se, standard error.
